# Differences in molecular phenotype in mouse and human hypertrophic cardiomyopathy

**DOI:** 10.1038/s41598-021-89451-6

**Published:** 2021-06-23

**Authors:** Styliani Vakrou, Yamin Liu, Li Zhu, Gabriela V. Greenland, Bahadir Simsek, Virginia B. Hebl, Yufan Guan, Kirubel Woldemichael, Conover C. Talbot, Miguel A. Aon, Ryuya Fukunaga, M. Roselle Abraham

**Affiliations:** 1 Division of Cardiology, Hypertrophic Cardiomyopathy Center of Excellence, Johns Hopkins University School of Medicine, Baltimore, MD USA; 2 Division of Cardiology, Hypertrophic Cardiomyopathy Center of Excellence, University of California San Francisco, San Francisco, CA 94158 USA; 3 Department of Biological Chemistry, Johns Hopkins School of Medicine, 725 N. Wolfe St, 521A Physiology, Baltimore, MD 21205 USA; 4 Intermountain Medical Center, Intermountain Heart Institute, Murray, UT USA; 5 Johns Hopkins School of Medicine, Institute for Basic Biomedical Sciences, Baltimore, MD USA; 6 Laboratory of Cardiovascular Science, National Institute on Aging/NIH, Baltimore, MD 21224 USA; 7 Present Address: National and Kapodistrian University of Athens, School of Medicine, Athens, Greece

**Keywords:** Genetics, Cardiology, Molecular medicine, Pathogenesis

## Abstract

Hypertrophic cardiomyopathy (HCM) is characterized by phenotypic heterogeneity. We investigated the molecular basis of the cardiac phenotype in two mouse models at established disease stage (mouse-HCM), and human myectomy tissue (human-HCM). We analyzed the transcriptome in 2 mouse models with non-obstructive HCM (R403Q-MyHC, R92W-TnT)/littermate-control hearts at 24 weeks of age, and in myectomy tissue of patients with obstructive HCM/control hearts (GSE36961, GSE36946). Additionally, we examined myocyte redox, cardiac mitochondrial DNA copy number (mtDNA-CN), mt-respiration, mt-ROS generation/scavenging and mt-Ca^2+^ handling in mice. We identified distinct allele-specific gene expression in mouse-HCM, and marked differences between mouse-HCM and human-HCM. Only two genes (*CASQ1, GPT1)* were similarly dysregulated in both mutant mice and human-HCM. No signaling pathway or transcription factor was predicted to be similarly dysregulated (by Ingenuity Pathway Analysis) in both mutant mice and human-HCM. Losartan was a predicted therapy only in TnT-mutant mice. KEGG pathway analysis revealed enrichment for several metabolic pathways, but only pyruvate metabolism was enriched in both mutant mice and human-HCM. Both mutant mouse myocytes demonstrated evidence of an oxidized redox environment. Mitochondrial complex I RCR was lower in both mutant mice compared to controls. MyHC-mutant mice had similar mtDNA-CN and mt-Ca^2+^ handling, but TnT-mutant mice exhibited lower mtDNA-CN and impaired mt-Ca^2+^ handling, compared to littermate-controls. Molecular profiling reveals differences in gene expression, transcriptional regulation, intracellular signaling and mt-number/function in 2 mouse models at established disease stage. Further studies are needed to confirm differences in gene expression between mouse and human-HCM, and to examine whether cardiac phenotype, genotype and/or species differences underlie the divergence in molecular profiles.

## Introduction

Hypertrophic cardiomyopathy (HCM) caused by mutations in genes encoding sarcomeric proteins^1^, is the most common cause of sudden cardiac death in young individuals^2–4^. These mutations lead to myocyte hypertrophy, disarray, interstitial/replacement fibrosis and arteriolar remodeling^5^, which underlie atrial/ventricular arrhythmias, heart failure and angina^6–8^.

Hypertrophic cardiomyopathy is characterized by phenotypic heterogeneity and variable penetrance. Two thirds of HCM patients have evidence of left ventricular (LV) obstruction at rest and/or exercise (obstructive or labile-obstructive HCM)^9^, which can be relieved by medications^10^, alcohol septal ablation or surgical myectomy^11^, whereas a third have no LV obstruction (non-obstructive HCM). Basic studies in human heart tissue obtained from genotype-positive and genotype-negative patients with obstructive or burned-out HCM demonstrate similarities in gene expression^12^ and high myofilament Ca^2+^ sensitivity^13^. Electrophysiology studies in myocytes from human myectomy tissue^14,15^ and mouse hearts^16^ indicate similarities in pro-arrhythmic remodeling of ion channel currents, CAMKII activation and abnormalities in cytoplasmic Ca^2+^ handling. But the association between genotype, cardiac phenotype and mRNA/miRNA expression has not been systematically investigated in  mouse and human HCM.

**Figure 1:**
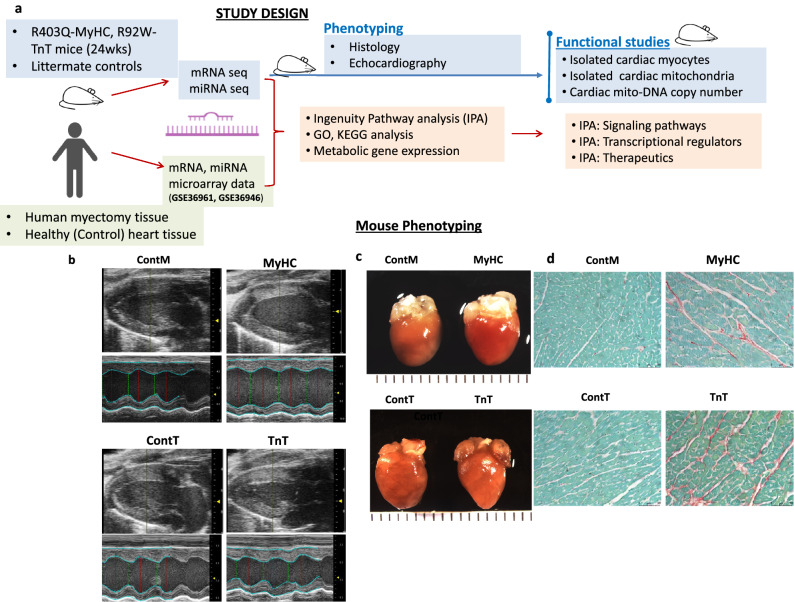
Mouse Phenotyping (**a**) Study design. (**b**) Echocardiography: Representative 2D and M-mode images from mutants (MyHC, TnT) and respective littermate control mice (ContM, ContT) illustrate that Left Ventricular (LV) cavity size is smaller in TnT-mutant mice, and larger in MyHC-mutant mice, when compared to littermate-controls. (**c**) Representative gross anatomy (left panel) and histology images (right panel) from mutants (MyHC, TnT) and littermate controls (ContM, ContT) reveal larger heart size in MyHC-mutants, and smaller heart size in TnT-mutants, when compared to littermate-controls. Bi-atrial enlargement is prominent in TnT-mutant mice. (**d**) Picrosirius Red staining reveals higher interstitial fibrosis in both mutant mouse hearts when compared to littermate-control hearts (for gross anatomy, calibration markers are 1 mm; microscopy calibration marker indicates 50 μm). For complete list of data with statistical analysis please see Table 1.

Using two mouse models carrying the R403Q mutation in the α-myosin heavy chain (MyHC)^17^ or the R92W mutation in cardiac troponin T (TnT)^18^, we have previously demonstrated allele-specific differences in gene expression, redox and mitochondrial function at an *early* stage of disease (5 weeks of age)^19^. It is unknown whether these differences persist in the setting of an *established cardiac HCM phenotype*, and to what extent gene expression overlaps in humans and mice at established disease stages. In order to address these questions, we analyzed cardiac gene expression in the same two mutant mouse models described above (R403Q-αMyHC, R92W-TnT) at an established stage of disease (non-obstructive HCM, 24 weeks of age), in parallel with heart tissue of patients with genotype-positive and genotype-negative obstructive HCM who underwent septal myectomy^20^ (Fig. 1a).

Here, we provide evidence for a distinct mRNA/miRNA biosignature at the *established* disease stage in two HCM mouse models. Both mutant mouse myocytes demonstrate evidence of an oxidized redox environment, abnormalities in mitochondrial complex I function and significant downregulation of several metabolic pathway genes. We further show differences in mRNA/miRNA expression, transcriptional regulation and signaling pathway dysregulation between the two mutant mice and human myectomy tissue. Taken together, our results highlight the need for parallel studies in mouse and human HCM to define the molecular basis of heterogeneity in cardiac HCM phenotype.

## Methods

An extended description of methods is provided in Supplemental materials. All methods were carried out in accordance with relevant guidelines and regulations.

### Experimental animals

All procedures involving the handling of animals were approved by the Animal Care and Use Committees of the Johns Hopkins University and the University of California San Francisco, and adhered to the National Institutes of Health Public Health Service guidelines. We studied 24 week old transgenic male C57Bl/6N mice expressing the R403Q mutation^17^ in αlpha myosin heavy chain gene *(Myh6 gene),* or the R92W mutation^18^ in the cardiac troponin T gene (*Tnnt2 gene),* along with littermate-controls (Fig. 1a)*.* Male mice were weaned and genotyped at 4 weeks of age using PCR-amplified tail DNA. Mouse euthanasia was performed by cervical dislocation, prior to harvesting hearts.

### Mouse echocardiography

All images were acquired using a Vevo 3100 imaging ultrasound machine and a MX550D probe (40 MHz, VisualSonics, Toronto, Canada) with ECG monitoring. Mice were anesthetized with isoflurane (2% for induction, < 1% during imaging) resulting in heart rates 500–550 bpm during cardiac imaging. Cardiac morphology and function were assessed from conventional M-mode and 2D images using Vevo LAB software.

### Human transcriptome data

The microarray datasets (GSE36961: mRNA, GSE36946: miRNA) in the NCBI GEO database were analyzed (Fig. 1a). RNA for the HCM group was obtained from ventricular septal tissue of HCM patients who underwent myectomy at the Mayo Clinic; Control group RNA was obtained from age/sex-matched donor hearts (LV septal or free wall) from the Sydney Heart Bank^21^; demographic and phenotype data was obtained from the Master’s Thesis by Virginia Hebl^20,22^. We note that the human myectomy dataset was generated from genotype-positive patients carrying several different sarcomeric protein gene mutations as well as genotype-negative individuals. Despite the genotype differences, we considered the human myectomy data as a single group (human-HCM) in our study, based on results of sub-group analysis^12^ which revealed few differences in cardiac gene expression between *MYH7/MYBPC3*-positive and genotype-negative HCM patients.

### Mouse transcriptome data

mRNA-seq libraries and miRNA-seq libraries were prepared using whole heart total RNA (3 biologic replicates) and sequenced on the HiSeq2500 (Illumina) platform and analyzed as described previously^23,24^. The SRA accession number for the mRNA-seq and miRNA-seq libraries reported in this manuscript is PRJNA559482**.**

### Cellular redox

Isolated cardiac myocytes^25^ were imaged by 2-photon microscopy at 37 °C to assess cellular redox status. Cell labeling with tetramethylrhodamine methyl ester (TMRM) and monochlorobimane (MCB) was used to simultaneously monitor mitochondrial membrane potential (Δψ_m_) and reduced glutathione (GSH) respectively^19^; NAD(P)H autofluorescence was monitored separately^19^. All cells were imaged in a quiescent (non-beating) state.

### Mitochondrial DNA copy number

This was assessed using qRT-PCR for *COX-1* and GAPDH as previously described^19,26^

### Isolated mitochondrial studies

Freshly isolated mitochondria were used to measure oxygen consumption rate (OCR) of individual electron transport chain respiratory complexes (ETC) using the Seahorse Biosciences Instrument. In addition, reactive oxygen species (ROS) generation/scavenging and Ca^2+^ handling was assayed by fluorometry, as described previously^19^.

### Statistics

All data are presented as mean ± SD unless otherwise noted. Fluorometry data was analyzed using Origin8 software. GraphPad Prism 8 was used for statistical analysis and design of graphs. The 2-tailed unpaired student’s t-test or the one sample t-test were used to compare each mutant to littermate controls, and to compare expression of selected genes from human HCM myectomy samples with control subjects. In order to facilitate comparison of data from the 2 mutants, data from mutants was normalized to data from respective littermate-controls and presented as log2fold change [log2FC=log2(expression value in mutants/expression value in littermate controls)]. For Ingenuity Pathway Analysis (IPA) of human samples, mRNA data from 105 HCM patients was compared to 39 controls by ANOVA using the Partek Genomics Suite 7.0 platform. A p < 0.05 was considered statistically significant.

## Results

### Cardiac phenotypic heterogeneity in mouse and human HCM at established disease stage

*Mouse HCM:* Echocardiography in 24-week-old mutant mice and littermate controls revealed that R403Q-MyHC mutant mice had higher left ventricular (LV) mass, lower LV ejection fraction (LVEF), but similar stroke volume (SV) when compared to littermate-controls, whereas R92W-TnT mutant mice had lower LV mass, similar LVEF but lower SV when compared to littermate-controls; none of the mice had LV outflow tract or mid-cavitary obstruction (Table 1, Fig. 1b,c). Picrosirius red staining revealed greater amount of interstitial fibrosis in both mutant mice when compared to littermate-controls (Table 1, Fig. 1d).

**Table 1:** Phenotypic characteristics of 24-week-old mutant and control mice.

Echocardiography	ContM	MyHC	P value	ContT	TnT	P value
Body weight (g)	28.1 ± 0.8	28.6 ± 1.5	0.5	27.1 ± 1.3	29.4 ± 2.6	0.5
Heart Rate (bpm)	517 ± 25	523 ± 22	0.4	521 ± 22	505 ± 30	0.08
LVESD (mm)	2.6 ± 0.2	2.9 ± 0.3	0.02	2.4 ± 0.4	2.1 ± 0.3	0.03
LVEDD (mm)	3.9 ± 0.2	4.1 ± 0.3	0.1	3.8 ± 0.4	3.5 ± 0.3	0.02
LVEF (%)	63 ± 5	57 ± 8	0.01	67 ± 7	71 ± 8	0.2
LVFS (%)	34 ± 4	29 ± 5	0.02	37 ± 5	39 ± 6	0.1
Stroke volume (µl)	43 ± 7	43 ± 7	0.8	44 ± 10	37 ± 7	0.03
LV mass (mg)	123 ± 14	139 ± 22	0.03	124 ± 29	108 ± 15	0.04
**Gross anatomy and histology**						
Heart weight (g)	0.13 ± 0.01	0.15 ± 0.01	0.03	0.12 ± 0.01	0.10 ± 0.05	0.03
HW/tibial length	0.070 ± 0.004	0.081 ± 0.006	0.03	0.065 ± 0.003	0.059 ± 0.003	0.006
Picrosirius red staining (%)	0.59 ± 0.00	1.94 ± 0.01	0.007	0.55 ± 0.00	1.82 ± 0.00	< 0.001

LV; left ventricular, LVESD; LV end systolic diameter, LVEDD; LV end diastolic diameter, LVEF; LV ejection fraction, LVFS; LV fractional shortening, HW; heart weight.

Two-sided unpaired student’s t-test was used for statistical comparisons between mutants and littermate controls (ContM vs MyHC and ContT vs TnT). For ECHO data, ContM (n = 15), MyHC (n = 20), ContT (n = 20), TnT (n = 18) were studied. For gross anatomy, n = 4 mouse hearts were included in each group. For histology, 20 randomly selected fields in the septum, apex and lateral wall were examined in each mouse heart (n = 2 mouse hearts in each group).

*Human HCM:* We analyzed publicly available microarray data from 107 patients and 39 healthy controls^12,27^. Genotype and phentype date was obtained from the publicly available Master thesis by Virginia Hebl. HCM patient age ranged from 9 to 78 years and 51% were male^22^; controls were age/sex matched. Genotyping (in 100/107 patients) revealed pathogenic variants in *MYBPC3* (n = 24), *MYH7* (n = 17), *TNNT2* (n = 4), *TNNC1* (n = 1), *TPM1* (n = 2), *MYL2* (n = 3), and *MYH6* (n = 1); no causal mutation was identified in 48 patients. Echocardiography revealed presence of LV hypertrophy (mean LV wall thickness of 2.2 ± 0.7 cm) and obstructive hemodynamics (mean LV gradient of 71.5 ± 47.4 mmHg) in the HCM group^22^.

### Transcriptomics in mouse-HCM and human-HCM

High-throughput sequencing of miRNAs (miRNA-seq) and mRNAs (mRNA-seq) from mouse hearts was performed to examine mutation-specific effects of the R403Q-MyHC and R92W-TnT mutations on global gene expression at an established disease stage (24 weeks of age).

#### miRNA profiling

We found no differences in miRNA expression between MyHC-mutant and littermate-control hearts (Fig. 2a,c,e–f). In contrast, expression of four miRNAs was significantly different in TnT-mutants compared to littermate-controls: miR-99b-5p, miR-195-5p^28^, miR-497a-5p were upregulated, and miR-150-5p^29,30^ was downregulated in TnT-mutants (Fig. 2b,d–g).

##### Mouse HCM (Fig. 2a–g):

**Figure 2:**
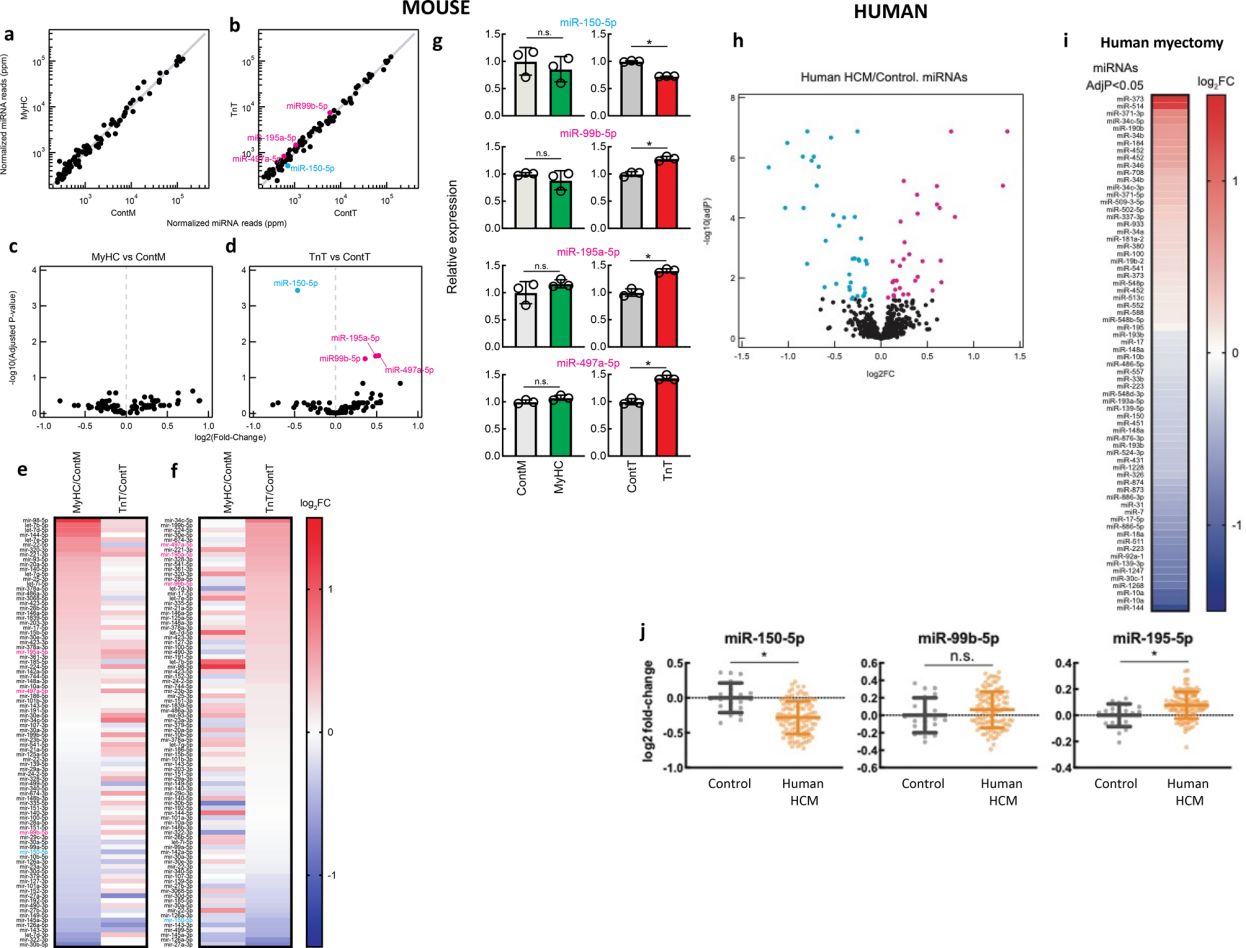
Global miRNA-seq analysis of MyHC-mutant, TnT-mutant mouse hearts and human-HCM. (**a**,**b**) Volcano plots of normalized miRNA reads in hearts of (**a**) MyHC-mutant mouse hearts, (**b**) TnT-mutant mouse hearts and littermate-control hearts (ContM and ContT). Each dot represents individual miRNA. The values are means of three biological replicates. Significantly (adjusted *p* < 0.05) upregulated miRNAs (miR-99b-5p, miR-195a-5p, miR-497a-5p) are shown in magenta and significantly downregulated miRNA (miR-150-5p) is shown in cyan in (**b**). miRNAs with > 300 normalized reads in any of the genotypes are shown. (**c**,**d**) Volcano plot of log2(fold-change) of miRNA-seq results in (**c**) MyHC-mutants and (**d**) TnT-mutants compared to littermate-controls. Benjamini–Hochberg method was used to adjust p values for multiple comparisons (n = 3 biological replicates). (**e**,**f**) Heatmap of log2(fold-change) of normalized miRNA reads in MyHC-mutants and TnT-mutants compared with littermate-controls (means of three biological replicates are presented). miRNAs are sorted in descending order of fold-change in abundance in (**e**) MyHC-mutants and (**f**) TnT-mutants. (**g**) Normalized miRNA abundance of miR-150-5p, miR-99b-5p, miR-195a-5p, and miR-497a-5p (mean ± S.D. n = 3 biological replicates; *p < 0.05 using 2-sided unpaired Student’s t-test). (**h**) Volcano plots of normalized miRNA reads in human myectomy tissue and control-hearts. (**i**) Heatmap of log2(fold-change) of normalized miRNA reads in human myectomy tissue (n = 107) compared to control-hearts (n = 20). miRNAs are ordered in descending order of fold-change in abundance (means are presented). (**j**) Normalized miRNA abundance of miR-150-5p, miR-99b-5p and miR-195a-5p (mean ± S.D.; n = 107 HCM patients/n = 20 controls; *p < 0.05 using 2-sided unpaired Student’s t-test).

##### Human HCM:

Among the 858 miRNAs that were analyzed on miRNA microarrays, 32 miRNAs were upregulated and 38 miRNAs were downregulated in myectomy tissue compared with control hearts (adjusted *p* < 0.05, FDR 5%) (Fig. 2h,i). Upregulated and downregulated miRNAs are shown in the heatmap (Fig. 2i). Of these 70 miRNAs, 2 miRNAs showed similar dysregulation in TnT-mutant mice and human myectomy tissue: miR-195-5p^28^ was upregulated and miR-150-5p^31^ was downregulated (Fig. 2j).

#### mRNA profiling

##### Mouse HCM:

**Figure 3:**
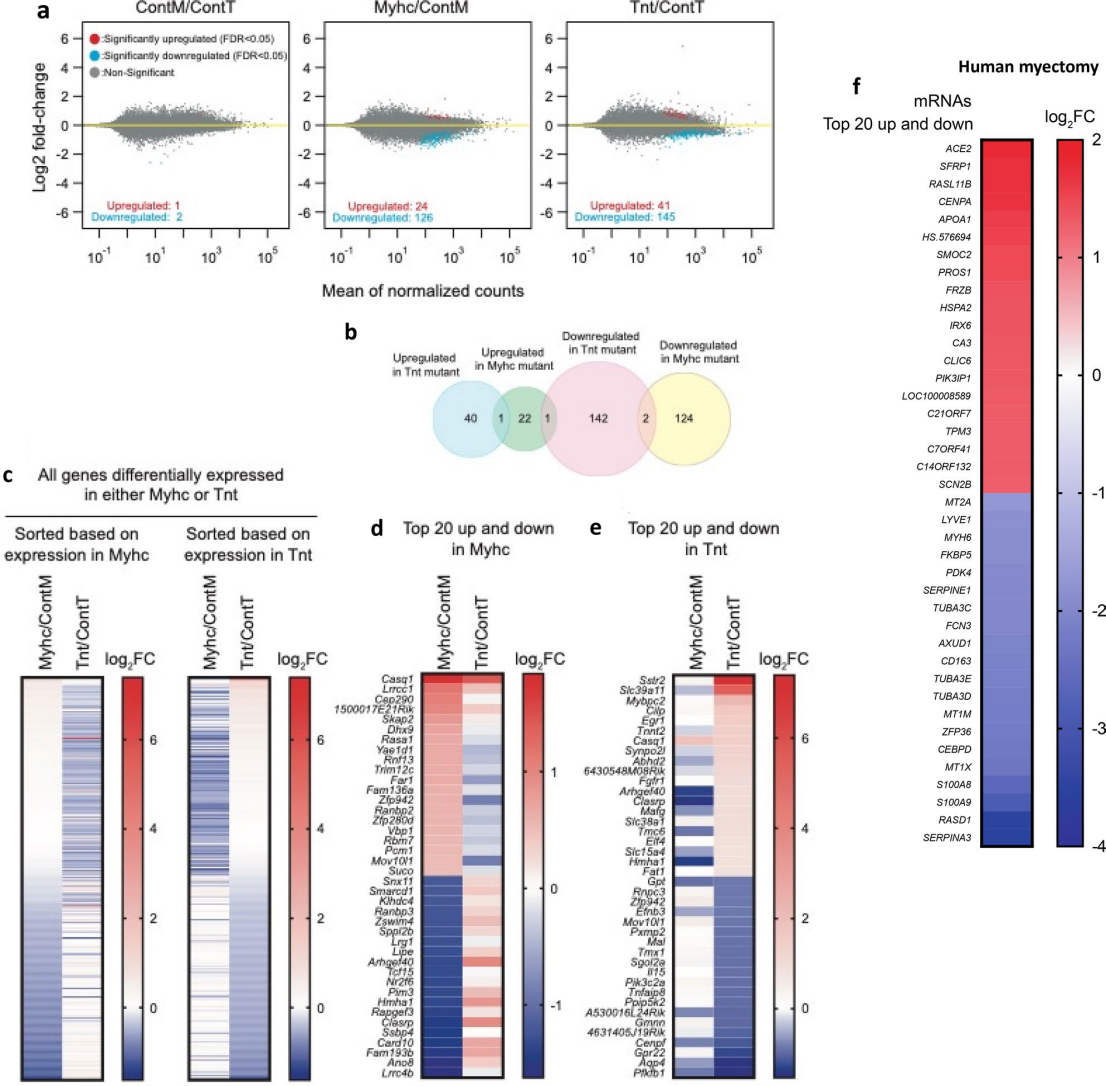
Global mRNA-seq analysis of MyHC and TnT-mutant mouse hearts and human-HCM. (**a**) Volcano plots of mRNA expression. X-axis shows mean of normalized counts among the 6 biological samples tested (3 biological replicates each for two genotypes compared in each graph) and Y-axis shows log2(fold-change) in the normalized counts. (**b**) Venn diagram of the numbers of differentially expressed genes. (**c**) Heatmaps of log2(fold-change) of mRNA expression levels in MyHC-mutant and TnT-mutant mouse hearts compared with littermate-control hearts for the 150 and 186 genes that are differentially expressed (adjusted *p* < 0.05) in either MyHC or TnT-mutant mice, respectively (means of three biological replicates are presented). The genes are sorted based on log2(fold-change) in MyHC-mutant mice (left panel) or in TnT-mutant mice (right panel). (**d**) Heatmap of log2(fold-change) of top 20 upregulated genes and downregulated genes in MyHC-mutant mice compared to littermate-controls (means of three biological replicates are presented). (**e**) Heatmap of log2(fold-change) of top 20 upregulated and downregulated genes in TnT-mutant mice compared to littermate-controls (means of three biological replicates are presented). (**f**) Heatmap of log2(fold-change) of top 20 upregulated and downregulated genes in human myectomy tissue compared to control hearts (means data is presented; n = 105 HCM patients/n = 39 controls).

Among the 24,228 annotated nuclear-encoded genes, only three mRNAs exhibited differential expression between the 2 control mouse hearts (ContM and ContT; adjusted *P* < 0.05, FDR 5%; Fig. 3a) whereas 150 mRNAs were significantly differentially expressed in MyHC-mutant hearts, and 186 mRNAs were significantly differentially expressed in TnT-mutant hearts (compared with littermate-control hearts; Fig. 3a,b). Among these differentially expressed mRNAs, only 3 overlapped and exhibited the same directional change between the two mutants: *Casq1 (*calsequestrin 1*)* was upregulated, whereas *Gpt1* (glutamic pyruvic transaminase) and *Rpl31* (ribosomal protein L31) were downregulated in both mutants. Heatmaps of mRNA levels for the differentially expressed genes show that MyHC-mutants and TnT-mutants have distinct gene dysregulation profiles (Fig. 3c–e, Supplemental Table 1). The most upregulated gene in MyHC-mutants was *Casq1* (3.6-fold), a Ca^2+^-storage protein expressed in the sarcoplasmic reticulum, whereas the most upregulated gene in TnT-mutants (1.7-fold) was *Sstr2* (somatostatin receptor 2), a negative regulator of β-adrenergic receptor signaling^32^.

##### Human HCM:

Among the 37,846 genes that were analyzed on the mRNA microarrays, 4202 genes were statistically significantly upregulated and 3,798 genes were downregulated in human myectomy tissue compared with control hearts (adjusted *p* < 0.05, FDR 5%). The most upregulated gene in the human-HCM dataset was *ACE2* (3.6-fold), a negative regulator of the renin–angiotensin–aldosterone system (Fig. 3f). In a prior study, we performed principle component analysis (PCA) of the mRNA data and showed clustering of HCM patient data, as well as good separation of patients from healthy controls in component 2 (Y-axis) of the PCA plot^27^.

*Comparison of transcriptomes in 2 mutant mice and human myectomy tissue revealed only two genes (calsequestrin 1, glutamic pyruvic transaminase) that are similarly dysregulated in mouse and human HCM.*

#### KEGG and GO analysis of mRNA data

Using the identified differentially expressed genes in the 2 mutant mouse hearts and human myectomy tissue, we performed KEGG pathway and Gene Ontology (GO) term enrichment analysis to identify molecular processes that are perturbed in the established disease stage of mouse-HCM and human-HCM.

##### Mouse HCM:

Both mutant mouse hearts demonstrated significant (p < 0.05) enrichment of several metabolic processes, namely, 4 metabolic processes in MyHC-mutants and 9 in TnT-mutants. Of these, ‘pyruvate metabolism’ was enriched in both mutants, whereas ‘fatty acid metabolism’, ‘valine, leucine, isoleucine degradation’ and ‘propanoate metabolism’ were only enriched in TnT-mutants. Other processes that were exclusively enriched in TnT-mutants include processes related to mitochondria (e.g. ‘electron transfer activity’, ‘NADH dehydrogenase complex’) (Table 2, Supplemental Table 2).

**Table 2:** List of select processes identified by KEGG^a^ and GO analysis in 24 weeks old mutant mice and human myectomy tissue.

R403Q-MyHC mouse heart	p value	R92W-TnT mouse	p value	Human myectomy tissue	p value
**KEGG**					
Endocrine and other factor-regulated calcium reabsorption	0.003	Val, Leu, Ile degradation	5.8E−6	Phagosome	3E−11
Pyruvate metabolism	0.01	Propanoate metabolism	0.001	Val, Leu, Ile degradation	9E−7
Glycerolipid metabolism	0.03	Fatty acid degradation	0.004		
N-Glycan biosynthesis	0.03	Pantothenate and CoA biosynthesis	0.006	Viral myocarditis	2E−5
Regulation of lipolysis in adipocytes	0.047	Insulin signaling pathway	0.01	Carbon metabolism	0.0002
		Pyruvate metabolism	0.02	Apoptosis	0.0006
		Cellular senescence	0.03	Propanoate metabolism	0.0006
		Fatty acid metabolism	0.04	HIF-1 signaling pathway	0.0007
		Phosphatidylinositol signaling system	0.04	Insulin resistance	0.001
		Insulin resistance	0.048	Pyruvate metabolism	0.002
**GO cellular component**					
Intrinsic component of organelle membrane	0.01	Mitochondrial matrix	3.4E−9	Contractile fiber	1.8E−9
Polysome	0.04	Oxidoreductase complex	4.8E−5	Cell-substrate junction	4.5E−9
		Mitochondrial protein complex	5.3E−5	Mitochondrial substrate	3.1E−5
		Organelle inner membrane	0.001	Actin cytoskeleton	7.2E−5
		Mitochondrial membrane part	0.02	ATPase complex	0.03
		NADH dehydrogenase complex	0.04	Mitochondrial inner membrane	0.03
**GO molecular function**					
ADP binding	0.006	Coenzyme binding	1.9E−4	Cofactor binding	4.1E−7
Single stranding DNA binding	0.01	Oxidoreductase activity on the CH-CH group of donors	0.0010	Cell adhesion molecule binding	1.5E−5
Basal transcription machinery binding	0.03	Electron transfer activity	0.002	Sulfur compound binding	1.5E−5
RNA polymerase II transcription factor binding	0.03	Oxidoreductase activity on the aldehyde or oxo group of donors	0.003	Oxidoreductase activity	1.9E−5
Ribonucleoprotein complex binding	0.04	Sulfur compound binding	0.004	Collagen binding	0.0001
Carboxylic ester hydrolase activity	0.04	Lipase activity	0.007	Electron carrier activity	0.0002
Catalytic activity acting on DNA	0.04	Carboxylic ester hydrolase activity	0.02	Oxidoreductase activity on CH-CH donors	0.0003
**GO biologic process**					
Neutral lipid metabolic process	3.9E−4	Fatty acid metabolic process	2.7E−6	Blood vessel morphogenesis	2.3E−9
Cell redox homeostasis	0.002	Small molecule catabolic process	1.2E−5	Negative regulation of growth	1.6E−8
Regulation of binding	0.005	Lipid modification	1.7E−5	Response to inorganic substance	4.4E−8
Glycerolipid metabolic process	0.006	Generation of precursors metabolites and energy	2.5E−5	Cofactor metabolic process	4.5E−8
DNA-templated transcription termination	0.008	Lipid catabolic process	1.6E−4	Actomyosin structure organization	8E−8
Response to temperature stimulus	0.01	Nucleoside biphosphate metabolic process	4.8E−4	Cellular metal ion homeostasis	1.2E−7
		Cellular amino acid metabolic process	0.002	Regulation of vasculature development	1.9E−7
		Organophosphate biosynthetic process	0.004	Plasma membrane organization	2E−7

Benjamini–Hochberg method was used to adjust p values for multiple testing.

^a^Kanehisa and Goto^64^, Kanehisa^65^, Kanehisa et al.^66^.

##### Human HCM:

In addition to 8 metabolic processes (including ‘valine, leucine, isoleucine degradation’, ‘pyruvate metabolism’), human myectomy tissue demonstrated significant enrichment of other processes including ‘phagosome’, ‘viral myocarditis’, ‘apoptosis’ (Table 2, Supplemental Table 2).

*Only one process, ‘pyruvate metabolism’ was enriched in both mutant mouse hearts and human myectomy tissue at the established disease stage.*

#### Metabolic gene expression

Since a significant proportion of enriched processes identified by KEGG pathway analysis are metabolic, we examined differential expression of metabolic pathway genes, with a focus on oxidative phosphorylation (OxPhos), Krebs cycle, fatty acid (FA), glucose and branched chain amino acid (BCAA) metabolism pathways, in mutant mouse hearts and human myectomy tissue.

##### Mouse HCM:

**Figure 4:**
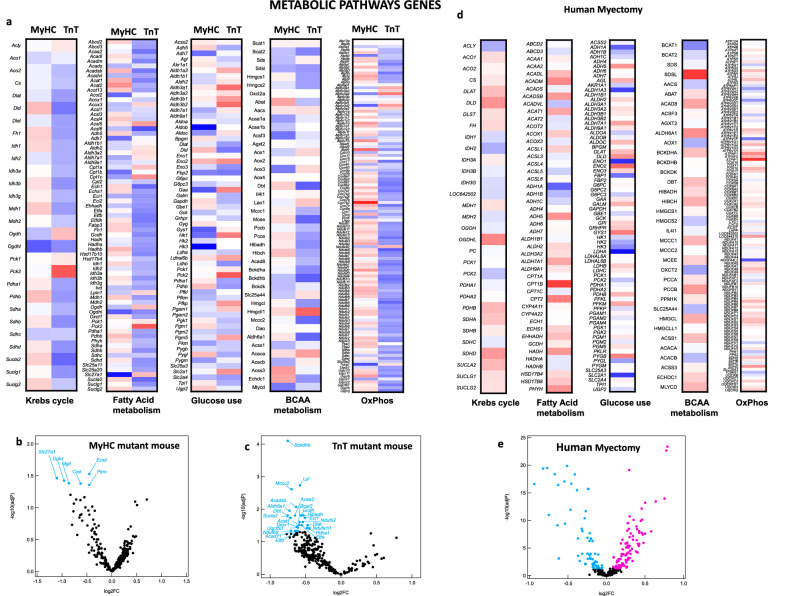
Differential metabolic gene expression in MyHC and TnT-mutant mouse hearts and human-HCM. (**a**) Heatmap of log2(fold-change) of normalized mRNA reads in MyHC-mutant and TnT-mutant mouse hearts compared with littermate-control hearts. mRNAs are clustered depending on the metabolic function of the involved metabolic genes (Krebs cycle, Fatty Acid metabolism, Glucose use, BCAA, OxPhos). Means of three biological replicates are presented. (**b**,**c**,**e**) Volcano plots of normalized mRNA reads of metabolic genes in MyHC-mutant and TnT-mutant mice compared to littermate-controls and in human myectomy tissue and control-hearts. Benjamini–Hochberg method was used to adjust p values for multiple comparisons; n = 3 biological replicates. (**d**) Heatmap of log2(fold-change) of normalized mRNA reads in human myectomy tissue compared to control-hearts (means are presented; n = 105 HCM patients/n = 39 controls). mRNAs are clustered depending on the metabolic function of the involved metabolic genes (Krebs cycle, Fatty Acids, Glucose use, BCAA, OxPhos).

Comparison of heat maps suggests differences in expression of genes involved in glycolysis, OxPhos, Krebs cycle, BCAA and FA metabolism pathways in the 2 mutant mouse hearts (Fig. 4a). Volcano plots revealed significant downregulation of 6 genes in MyHC-mutants (log2FC: − 0.45 to − 1.11), and 22 genes in TnT-mutants (log2FC: − 0.47 to − 0.75) when compared to littermate-controls (Fig. 4b,c, Supplemental Table 3). The most downregulated genes were *Slc27a1* (log2FC: − 1.11) in MyHC-mutants, and *Bckdhb, Dbt* (log2FC − 0.75) in TnT mutants; *Slc27a1* is involved in ATP-dependent import of long-chain FA into cells, whereas *Bckdhb, Dbt* are involved in BCAA catabolism.

##### Human HCM:

Human myectomy tissue demonstrated significant downregulation (n = 51 genes; log2FC − 0.09 to − 0.94) and upregulation (n = 90 genes; log2FC: 0.09 to 0.75) of several metabolic genes involved in OxPhos, pyruvate, FA, glucose and AA metabolism (Fig. 4d,e, Supplemental Table 4). The most down and up regulated metabolic genes respectively, were lactate dehydrogenase A (*LDHA*, log2FC: − 0.95) a glycolytic enzyme, and carnitine palmitoyltransferase 1B (*CPT1B*, log2FC: 0.75) which is involved in FA transport into mitochondria, a rate limiting step in FA oxidation.

*In summary, our analysis of global mRNA and miRNA expression revealed distinct gene expression patterns in the two mutant mouse hearts and in human myectomy tissue.*

### Allele-specific differences in mitochondrial number, function and cellular redox in mouse-HCM

Since HCM mutant mice demonstrated differences in expression of metabolic and electron transport chain (ETC) genes, we hypothesized the presence of differences in cardiac mitochondrial function in the two mutant mice, at established disease stage. To test this hypothesis, we examined cardiac mitochondrial DNA copy number (mtDNA-CN), respiration, reactive oxygen species (ROS) generation/scavenging in MyHC/TnT-mutant mice and littermate-controls at 24 weeks of age. Additionally, since redox status influences several cellular processes, including gene expression, protein function, excitation–contraction coupling and energetics^33^, we also examined myocyte redox in both mutant mice and littermate-controls at 24 weeks of age. (We did not have access to human myectomy tissue and hence were unable to examine mitochondrial number/function and redox in human-HCM).

#### Whole mouse hearts:

**Figure 5:**
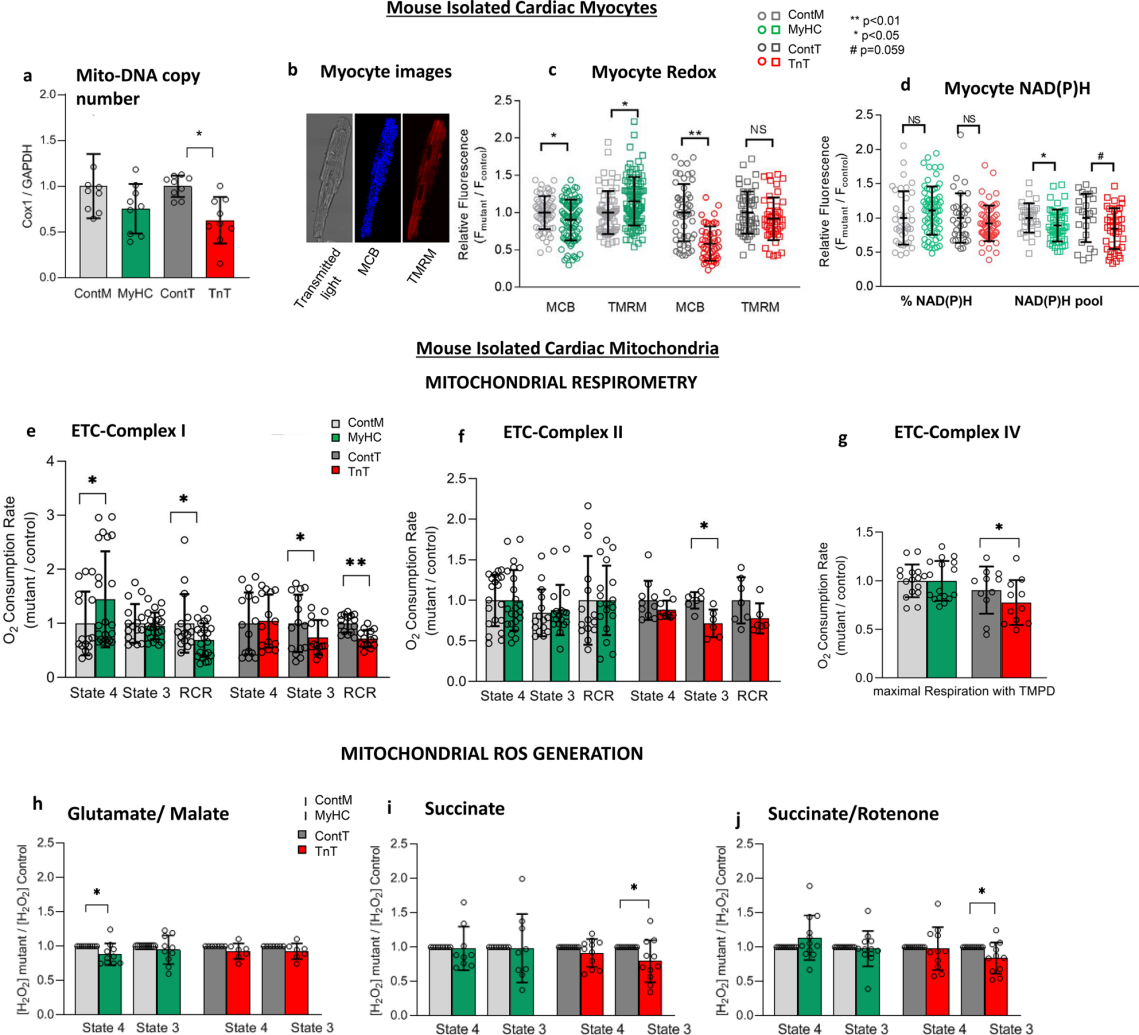

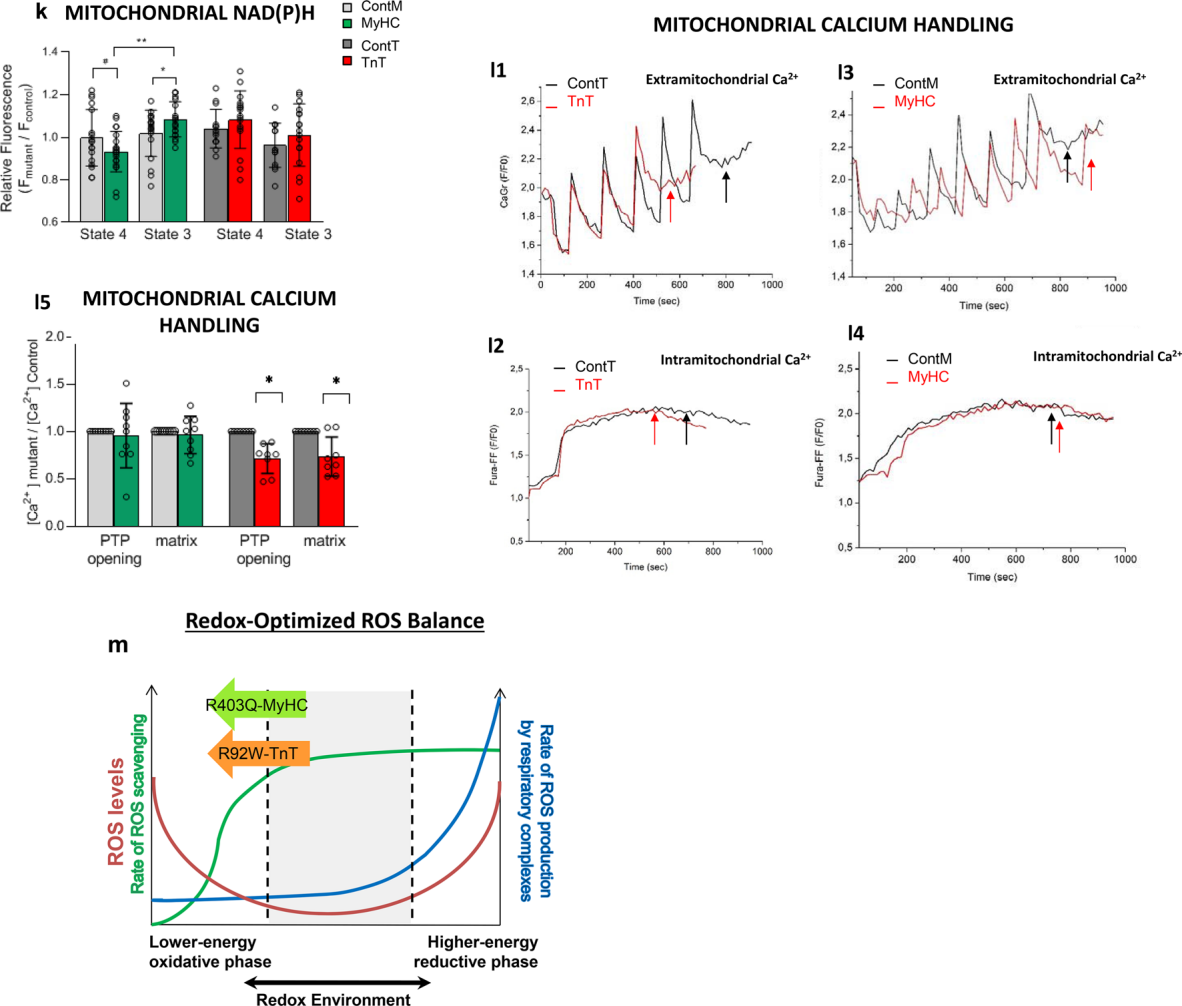
Myocyte and mitochondrial studies in mutant and littermate control mouse hearts. (**a**) Mitochondrial DNA copy number (mtDNA-CN) is presented as relative copy number of COX/GAPDH. MtDNA-CN in mutants was normalized to littermate-control data. MyHC-mutants had similar mtDNA-CN, whereas TnT-mutants had lower mtDNA-CN when compared to littermate-controls (n = 9 hearts in each group; *p = 0.02 using 2-sided unpaired student’s t-test). (**b**–**d**) Two-photon microscopy: (**b**) Representative images of cardiac myocytes. From right to left, unstained myocyte (transmitted light) and stained with MCB (*blue*) and TMRM (*red*) to simultaneously monitor reduced glutathione (GSH) and mitochondrial membrane potential (Δψ_m_) respectively. (**c**) Both mutant mouse myocytes had lower levels of GSH when compared to controls. ΔΨ_m_ was more hyperpolarized in MyHC-mutants, but similar in TnT-mutants, when compared to littermate-controls. (**d**) NAD(P)H levels were similar, but NAD(P)H pool levels were lower in both mutants when compared to littermate-controls (n = 3 mice in each group with ≥ 30 cells imaged per animal; *p < 0.05, **p < 0.01 using 2-sided unpaired student’s t-test. Fluorescence unit (FU) data is normalized to littermate-controls. %NAD(P)H was calculated based on NAD(P)H FU, normalized to respective NAD(P)H pool). (**e**–**g**) Mitochondrial respiration: (**e**) Complex I respiration: MyHC-mutant mitochondria had higher state 4 respiration, whereas TnT-mutants had lower state 3 respiration resulting in lower complex I respiratory control ratio (RCR) in both mutants when compared to littermate-controls. (**f**) Complex II respiration: Only TnT-mutants had lower state 3 respiration compared to littermate-controls. (**g**) Complex IV respiration: Only TnT-mutants exhibited lower complex IV respiration when compared to littermate-controls (n = 8 mice in ContM and TnT-mutants; n = 10 mice in ContT; n = 12 mice in MyHC-mutants; *p < 0.05, using 2-sided unpaired student’s t-test). (**h**–**j**) Mitochondrial H_2_O_2_ (ROS) generation: MyHC-mutant mitochondria demonstrated lower ROS during state 4 with glutamate/malate but similar ROS during State 3 as littermate-controls. MyHC-mutants showed similar ROS with succinate ± rotenone. TnT-mutants demonstrated lower ROS emission in state 3 with succinate ± rotenone (n = 8 mice for ContT and TnT-mutants; n = 10 mice for ContM and MyHC-mutants; *p < 0.05, using 2-sided one sample t-test). (**k**) Mitochondrial NAD(P)H: MyHC-mutant mitochondria had lower levels of reduced NAD(P)H during state 4, but higher levels during state 3 compared to littermate-controls; TnT-mutants had similar reduced NAD(P)H in state 3/4 as littermate-controls (n = 10 mice for ContT; n = 12 mice for ContM and TnT-mutants; n = 14 mice for MyHC-mutants. *p < 0.05, **p < 0.01 using 2-sided unpaired student’s t-test). (**l**) Mitochondrial Ca^2+^ handling: Mitochondria were pre-incubated with Fura-FF (20 μΜ) to monitor [Ca^2+^] changes in the mitochondrial matrix. Calcium Green-5N (0.1 μM) was added at the beginning of the experiment to monitor extra-mitochondrial [Ca^2+^] changes. Mitochondria were energized with glutamate/malate (GM) and additions of CaCl_2_ followed until the opening of PTP occurred (reflected by abrupt slow increase in the extra-mitochondrial calcium signal and slow decrease in the intra-mitochondrial calcium signal. Traces of extramitochondrial (l1, l3) and intramitochondrial (l2, l4) calcium signal in mutant TnT or MyHC (red trace) and littermate control (black trace) mitochondria. l5. Bar graphs summarizing mitochondrial Ca^2+^ handling Mitochondrial permeability transition pore (mPTP) activation occurred at similar [Ca^2+^] as littermate-controls in MyHC-mutants, but at lower [Ca^2+^] than littermate-controls in TnT-mutants. When compared to littermate-controls, matrix [Ca^2+^]_free_ was similar in MyHC-mutants and lower in TnT-mutants (n = 10 mice for MyHC-mutants/controls; n = 8 mice for TnT-mutants/controls; *p < 0.05, using 2-sided one sample t-test). *(**a**–**l**): *all comparisons were made for each group (ContM vs MyHC and ContT vs TnT)*. (**m**) Redox-optimized ROS balance: Both mutant mouse hearts are characterized by a lower-energy oxidized redox environment, due to lower reduced GSH and NAD(P)H pool levels, despite lack of increase in mitochondrial ROS generation.

Based on previous studies which demonstrated an association between reduction in mtDNA-CN and mitochondrial dysfunction^34,35^, we measured mtDNA-CN, which regulates expression levels of 13 proteins involved in OxPhos, 22 tRNA and 2 rRNA subunits in mitochondria^36^. TnT-mutant hearts had significantly lower mtDNA-CN, whereas MyHC-mutant hearts had similar mtDNA-CN when compared to littermate-controls (Fig. 5a).

#### Isolated mouse cardiac myocytes:

Cardiac myocytes were labeled with TMRM to evaluate mitochondrial membrane potential (Δψ_m_), and MCB to measure GSH levels (Fig. 5b). Cellular autofluorescence was used to assess cellular NAD(P)H; NAD(P)H signal was calibrated by the addition of KCN for maximum reduction, followed by FCCP for maximum oxidation of NADPH^19,25,26^. MyHC-mutant myocytes demonstrated a more hyperpolarized Δψ_m_, whereas TnT-mutants had similar Δψ_m_ when compared to littermate-controls (Fig. 5c). NAD(P)H pool levels and GSH were lower in both mutants when compared to littermate-controls (Fig. 5d), reflecting an oxidized redox environment in cardiac myocytes of both mutant mice at the established disease stage.

#### Isolated mouse mitochondria

##### Respirometry

We assessed function of ETC complexes I, II, and IV in isolated cardiac mitochondria by respirometry (Seahorse XF96 bioanalyzer). Oxygen Consumption Rate (OCR) was measured using glutamate/malate, succinate (with rotenone) and ascorbate/TMPD which are substrates of complexes I, II and IV respectively, in state 4 (absence of ADP) and state 3 (presence of ADP). Coupling of OxPhos was assessed by computing the Respiratory Control Ratio (RCR = state 3/state 4 respiration)^19,25,26^. Complex I RCR was lower in both mutant mitochondria, due to higher state 4 respiration in MyHC-mutants, and lower State 3 respiration in TnT-mutants, when compared to littermate-controls (Fig. 5e). Only TnT-mutant mitochondria had lower complex II-state 3 respiration, and lower complex IV respiration, when compared to littermate-controls (Fig. 5f,g).

##### Mitochondrial redox

Mitochondrial oxidative metabolism leads to generation of ROS, which serve as signaling molecules under physiologic conditions as well as pathologic states such as cardiac hypertrophy. Superoxide produced by mitochondria is rapidly dismutated to H_2_O_2_ which can be quantified by Amplex Red^26^. We used fluorometry to measure H_2_O_2_ generation/scavenging and NAD(P)H redox status in isolated mitochondria^19,26^.

*ROS (H*_*2*_*O*_*2*_*) generation and scavenging in isolated mitochondria*: In order to investigate the contribution of mitochondrial ROS to the oxidized redox environment observed in mutant mouse myocytes, we measured ROS generation and scavenging in isolated mitochondria. ROS generation was measured in the presence of complex I substrates glutamate/malate (NADH-oxidizing, forward electron transport), complex II substrate succinate (reverse electron transport to complex I), and rotenone (complex I inhibitor) plus succinate^19,26^.

Glutamate/malate oxidation in the presence of ADP (state 3) resulted in similar ROS levels in both mutant-mitochondria, compared to littermate-controls. MyHC-mutants had lower ROS generation with glutamate/malate in state 4, but similar ROS generation as littermate-controls in the presence of succinate and/or rotenone during states 4 and 3. TnT-mutants demonstrated lower ROS generation during state 3, in the presence of succinate and/or rotenone, but similar ROS during state 4 (Fig. 5h–j).

The thioredoxin (Trx) and glutathione (GSH) systems are the main ROS scavengers in the mitochondrial matrix, with NADPH being the main electron donor for these two systems. We used Auranofin, a selective inhibitor of thioredoxin reductase 2 (TrxR2), and 1-chloro-2,4-dinitrobenzene (DNCB), a GSH depleting agent, to assess ROS scavenging capacity of mutant and littermate-control mitochondria during states 4 and 3^19,37^. Both mutant mitochondria exhibited similar ROS scavenging capacity by the Trx and GSH systems, as littermate-controls, during state 4 and 3 respiration (Supplemental Fig. S1).

*Mitochondrial NAD(P)H*: The NAD(P)H signal was monitored in isolated mitochondria during state 4 and state 3 respiration, in the presence of complex I substrates, glutamate/malate; the NAD(P)H signal was calibrated by the addition of KCN (2.5 mM) for maximal reduction, and 2,4-dinitrophenol (DNP, 20 μM) for maximal oxidation^19,26^. MyHC-mutant mitochondria had lower NAD(P)H levels during State 4, but higher NAD(P)H levels during State 3 when compared to littermate-controls, which could be secondary to lower mitochondrial NAD(P)H levels and lower NAD(P)H oxidation. We found no difference in mitochondrial NAD(P)H levels in state 4 and state 3 between TnT-mutants and littermate-controls (Fig. 5k).

##### Mitochondrial calcium handling

Mitochondria are an important Ca^2+^ sink in cardiac myocytes. Additionally, mitochondrial-Ca^2+^ regulates energetics by stimulating three Krebs cycle dehydrogenases and mitochondrial ATP-synthase. Mitochondrial Ca^2+^ overload triggers mitochondrial permeability transition pore (mPTP) opening which can result in mitochondrial dysfunction and cell death. In order to assess whether differences in mitochondrial Ca^2+^ handling detected in early disease stage in mutant mice^19^, also persist at the established disease stage, we measured extra-mitochondrial and intra-mitochondrial [Ca^2+^] simultaneously by fluorometry^38^, using Calcium Green-5N and Fura-FF, respectively (Fig. 5l1–14). Mitochondrial Ca^2+^ handling was similar in MyHC-mutants/littermate-controls. In contrast, TnT-mutants demonstrated lower matrix [Ca^2+^]_free_ and higher sensitivity to Ca^2+^, evidenced by mPTP opening at lower [Ca^2+^] uptake, when compared to littermate-controls (Fig. 5-l5).

*In summary, our studies in isolated mitochondria and myocytes, viewed in the context of the redox-optimized ROS balance hypothesis*^26^*, suggest that both mutant mouse hearts lie on the oxidized side of the redox environment at the established disease stage (Fig. *5*m*).

### Ingenuity Pathway Analysis

Since metabolites and ROS can influence intracellular signaling and transcriptional networks, we interrogated dysregulated signaling pathways and transcriptional regulators in the two mutant mouse hearts and human myectomy tissue, using Ingenuity Pathway Analysis (IPA) of mRNA data. We used a cutoff value for significance of p < 0.01 and |z-score|> 1.

#### Predicted signaling pathways in mouse-HCM and human-HCM

**Figure 6:**
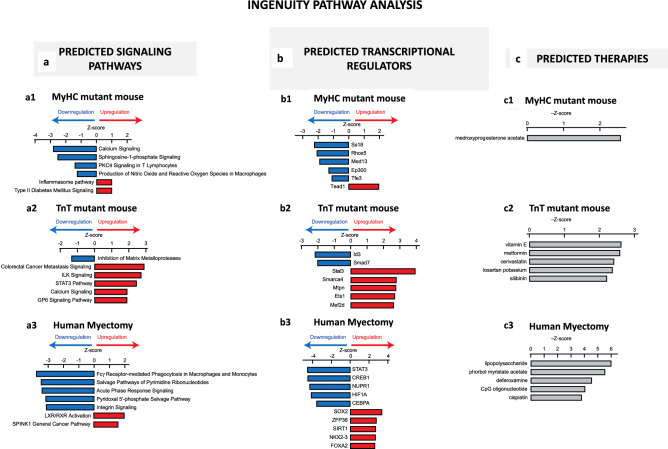
Ingenuity pathway analysis: top 5 dysregulated signaling pathways (**a**), transcriptional factors (**b**) and therapies (**c**) are presented for MyHC-mutant (1) TnT-mutant (2) mouse hearts, and human myectomy tissue (3). All comparisons are made to corresponding controls. Cutoff value for significance of *p* < 0.01 and |Z-score|> 1. Comparison of mRNA data from mutant HCM mice and human myectomy samples with respective controls was performed by ANOVA using the Partek Genomics Suite 7.0 platform.

In MyHC-mutant mouse hearts, the inflammasome pathway^39^ and type 2 diabetes mellitus signaling were the only two pathways predicted to be upregulated, whereas calcium signaling was downregulated (Fig. 6a1; Supplemental Fig. S2a). In contrast, TnT-mutant mouse hearts had predicted upregulation of several pathways including calcium signaling^40^ and STAT3 (signal transducer and activator of transcription 3)^41,42^; only one pathway (inhibition of matrix metalloproteases) was predicted to be downregulated in TnT-mutant mice (Fig. 6a2; Supplemental Fig. S2b).

The anti-hypertrophic/anti-fibrotic LXR/RXR (liver X receptor/retinoid X receptor) pathway was the most upregulated pathway in human myectomy tissue. A large number of pathways were predicted to be downregulated, including STAT3^41,42^ and pro-hypertrophic endothelin-1 (ET-1) signaling (Fig. 6a3; Supplemental Fig. S2c).

*No signaling pathway was predicted to be similarly dysregulated in both mutant mouse hearts and in human myectomy tissue*.

#### Predicted transcriptional regulators in mouse-HCM and human-HCM

IPA predicted upregulation of TEAD1 as well as downregulation of two pro-hypertrophic transcription factors/TFs (EP300^43^, TFE3), and MED13, a regulator of systemic energy homeostasis^44^ in MyHC-mutant mouse hearts. In contrast, TnT-mutant mouse hearts had predicted upregulation of several pro-hypertrophic TFs (GATA4, MEF2D, MITF, SMARCA4^45^, MTPN, SRF) and pro-fibrotic ETSI, along with downregulation of anti-fibrotic SMAD7^46^ (Fig. 6b1-2; Supplemental Fig. S3a,b).

In human myectomy tissue, IPA predicted upregulation of nine transcription factors (TF), including one cardiac TF (NKX2-3), three TFs that regulate metabolism (SOX2, MED13, SIRT1), and two TFs that inhibit TGF-β signaling/fibrosis (SMAD7, DACH1). A large number of TFs were predicted to be downregulated, including pro-hypertrophic NFATC2, pro-fibrotic SMAD1-4, as well as TFs that regulate metabolism/mitochondrial function (HIF1α, CREB1) (Fig. 6b3; Supplemental Fig. S3c).

*No transcription factor was predicted to be similarly dysregulated in both mutant mouse hearts and in human myectomy tissue (Supplemental Fig. *2).

#### Predicted therapies in mouse-HCM and human-HCM

Since IPA predicted marked differences in signaling pathways and transcriptional regulators in both mutant mouse hearts and human myectomy tissue, we hypothesized the need for individualized therapies. Using IPA we found that predicted therapies were markedly different in both mutant mice and in human-HCM (Fig. 6c1–3; Supplemental Fig. S4a–c). Losartan, a clinically used angiotensin receptor blocker was a predicted therapy for TnT-mutant mice, but not MyHC-mutant mice or human-HCM.

*In summary, our results suggest unique transcriptional regulation and potential benefit of tailored therapies in the established disease stage of hypertrophic cardiomyopathy.*

## Discussion

This study examined the molecular basis of phenotypic heterogeneity in HCM, by examining global cardiac gene expression in parallel, in two HCM mouse models and human myectomy tissue. Using a combination of heart imaging and unbiased methods, we demonstrate distinct molecular bio-signatures in two HCM mouse models with non-obstructive HCM at the established disease stage, and in human myectomy tissue obtained from genotype-positive and negative patients with obstructive HCM.

Our findings of marked differences in gene expression between two HCM mouse models at established disease stage are in contrast to results of genotype-based subgroup analysis performed by Bos et al.^12^ (using this human myectomy dataset), which revealed few differences in cardiac gene expression between patient subgroups. This discrepancy (namely, allele-specific differences in gene expression in mouse-HCM but not in human-HCM) could result from marked differences in cardiac physiology between HCM patients and our 2 mouse models at the established disease stage. Notably, the mouse hearts did not have evidence of LV obstruction, in contrast to the HCM patients who had severe LV obstruction necessitating septal myectomy for relief of obstruction and symptoms. We hypothesize that the effect of severe LV obstruction on cardiac structure/function and gene expression, may have overshadowed mutation-driven differences in gene expression, resulting in a rather homogenous pattern of gene expression in patients with obstructive HCM. Studies in non-obstructive HCM patients are needed to ascertain whether mutation-specific differences in gene expression, mitochondrial/myocyte physiology observed in HCM mouse models are also present in human-HCM.

### Metabolic pathway genes

Both mutant mouse hearts demonstrated significant downregulation of several metabolic genes (N = 6 genes in MyHC mutants; N = 22 genes in TnT mutants), whereas human myectomy tissue demonstrated both up and downregulation of several metabolic genes (Supplemental Tables 3, 4). Only TnT-mutant mouse hearts demonstrated significant downregulation of genes encoding proteins involved in the Krebs cycle, electron transport chain and the adenine nucleotide tranlocator (Supplemental Tables 3, 4). We also observed upregulation of miR-195-5p expression in TnT-mutant hearts and human myectomy tissue. miR-195-5p is considered a metabolic miR because it can regulate expression of SIRT1^47^ and SIRT3^28^. Studies in patients with advanced heart failure and experimental models of heart failure reveal that miR195-5p regulates activity of key mitochondrial metabolic pathways such as Krebs cycle, FA oxidation, OxPhos as well as mitochondrial ROS scavenging, by reducing SIRT3-mediated deacetylation of mitochondrial proteins^28^. We hypothesize that downregulation of genes involved in energy metabolism combined with post-translational modifications that reduce enzyme activity could lead to energetic stress^48^ and arrhythmias^4,49^, which are a prominent clinical feature of patients expressing the R92W-TnT mutation.

In our study, TnT-mutant mouse hearts had significant downregulation of 6 genes involved in BCAA catabolism, whereas no difference was observed in MyHC-mutants. Human myectomy tissue had significant downregulation (n = 8) and upregulation (n = 24) of several BCAA pathway genes (Supplemental Tables 3, 4). Suppression of BCAA catabolism has been reported in human dilated cardiomyopathy and experimental models^50^, leading to the hypothesis that BCAA catabolic reprogramming is a compensatory response of the myocardium to stress, permitting redirection of amino acids to protein synthesis and cell growth during cardiac hypertrophy^50^. Metabolic flux studies and proteomics are needed to assess whether the changes in metabolic gene expression observed in our study are associated with reduction in OxPhos, Krebs cycle flux, BCAA catabolism and FA metabolism in HCM.

### Redox Status

Our results in isolated myocytes and mitochondria lead us to posit that both mutant mouse hearts reside in the lower-energy oxidative phase of the “redox axis” (Fig. 5m). Under these conditions, the ROS imbalance is driven by decreased antioxidant capacity rather than increased ROS generation. Our data show that the lower ROS scavenging results from decreased levels of the cellular pool of GSH and NAD(P)H in both mutant myocytes when compared to littermate-controls. We did not pace cells, and also did not measure extramitochondrial ROS generation by NADPH oxidase, nitric oxide synthase and/or xanthine oxidase. Since an oxidized redox environment can impair energetics^51^, parallel assessment of intra-mitochondrial and cytosolic ROS generation/scavenging with cell pacing are needed to understand the mechanism(s) underlying the oxidized redox environment and its impact on cardiac phenotype.

We obtained lists of genes involved in ROS generation and scavenging processes in mouse and humans using the web application AmiGO^52^ (Supplemental Tables 5, 6). We did not find statistically significant dysregulation of redox-related genes in MyHC-mutant mouse hearts, but TnT-mutants had modest downregulation of *Sod2* (mitochondrial superoxide dismutase, log2FC: − 0.47), *Gstm7* (glutathione S-transferase-Mu7, log2FC: − 0.69), and upregulation of *Cygb* (cytoglobin, log2FC: 0.7). In contrast, human myectomy tissue demonstrated dysregulation of 35 redox-related genes (Supplemental Fig. S5a–d, Supplemental Table 6). The most dysregulated redox gene in human myectomy tissue was *S100 calcium-binding protein A9* (*S100A9*, log2FC: − 2.91)^53^ which has been shown to induce NADPH oxidase 1 (NOX1) and modulate inflammation^54^.

### Cardiac Hypertrophy and Fibrosis

Since MyHC-mutant mice have higher LV mass, and TnT-mutant mouse hearts have lower LV mass when compared to littermate-controls (Table 1), we expected greater upregulation of pro-hypertrophic signaling pathways and TFs in MyHC-mutants. But to our surprise, IPA predicted activation of several pro-hypertrophic TFs and signaling pathways in TnT-mutant mouse hearts but not in MyHC-mutants (Supplemental Figs. S2, S3). Similarly, heart tissue from HCM patients with significant LV septal hypertrophy undergoing myectomy exhibited upregulation of anti-hypertrophic/anti-fibrotic genes/TFs/pathways (*ACE*2, LXR/RXR, SMAD7), and downregulation of pro-hypertrophic/pro-fibrotic TFs/signaling pathways (ET1, NFAT, STAT3, SMAD1-3 (Supplemental Figs. S2, 3).

**Figure 7:**
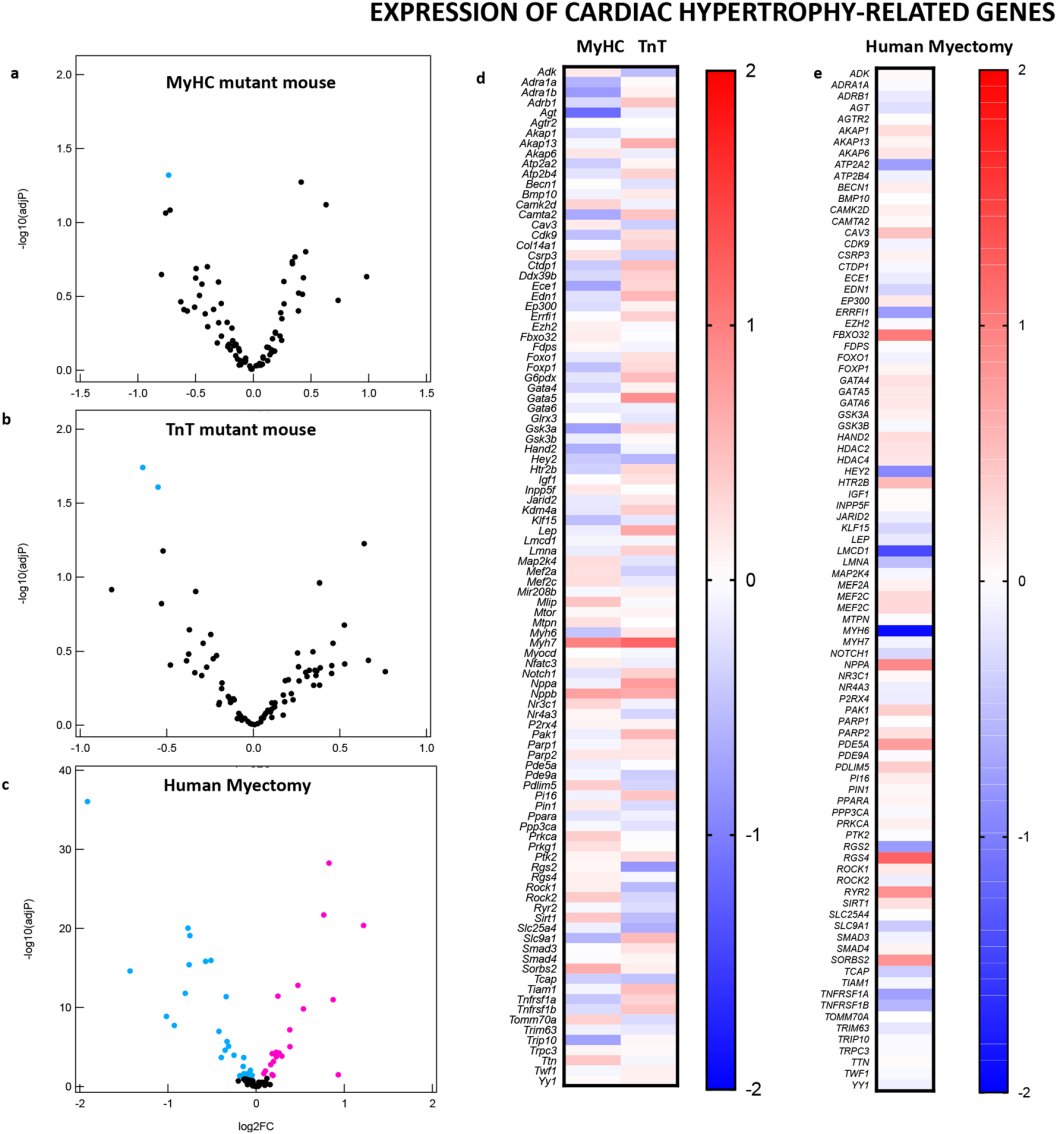
Genes implicated in cardiac hypertrophy and its regulation: volcano plots of normalized mRNA reads in MyHC and TnT-mutant mouse hearts (**a**,**b**) and human myectomy tissue (**c**). Heatmap of log2(fold-change) of differentially expressed genes related to hypertrophy and its regulation in MyHC-mutant and TnT-mutant mouse hearts (**d**) and in human myectomy tissue (**e**) compared to respective controls. Benjamini–Hochberg method was used to adjust p values for multiple comparisons; n = 3 biological replicates for mouse data; n = 105 HCM patients/n = 39 controls for human data). Genes involved in TGF-β signaling pathway and its regulation: Volcano plots of normalized mRNA reads of TGF-β pathway related genes in MyHC-mutant and TnT-mutant mouse hearts (**a**,**b**) and human myectomy tissue (**c**). Heatmap of log2(fold-change) of differentially expressed genes related to TGF-β signaling pathway and its regulators in MyHC-mutant and TnT-mutant mouse hearts (**d**) and in human myectomy tissue (**e**) compared to respective controls. Benjamini–Hochberg method was used to adjust p values for multiple comparisons (n = 3 biological replicates for mouse data; n = 105 HCM patients/n = 39 controls for human data).

Since the cardiac TFs^55^ and signaling pathways commonly associated with pathologic cardiac hypertrophy^56^ were not predicted to be upregulated by IPA, we searched for genes involved in cardiac hypertrophy and its regulators using AmiGO (Supplemental Tables 5–7). For cardiac hypertrophy, we identified 3 statistically significant dysregulated genes in mice and 42 dysregulated genes in human myectomy tissue. *Trip10* (thyroid hormone receptor interactor 10, log2FC: − 0.73) was significantly downregulated in MyHC-mutant mouse hearts; *Rock1* (log2FC: 0.12) implicated in perivascular fibrosis, transition from hypertrophy to heart failure, and *Slc25a4* (adenine nucleotide transporter, log2FC: − 0.13) were significantly downregulated in TnT-mutant mouse hearts (Fig. 7a,b,d, Supplemental Table 5). The most dysregulated cardiac hypertrophy-related gene in human myectomy tissue was *MYH6 (*alpha myosin heavy chain*,* log2FC: − 1.91) (Fig. 7e, Supplemental Table 7).

Interstitial and replacement fibrosis are frequently observed in HCM patients undergoing myectomy or heart transplantation. In contrast, both our mouse models demonstrated interstitial but not replacement fibrosis at the established disease stage. We observed downregulation of anti-fibrotic miR-150-5p in both human myectomy tissue and TnT-mutant hearts, but not in MyHC-mutants. miR-150-5p inhibits activation of cardiac fibroblasts^29^, and has been identified as a circulating biomarker of maladaptive cardiac remodeling and disease severity in patients with advanced heart failure^57^. Downregulation of miR-150-5p could be a contributor to development of cardiac fibrosis in HCM.

**Figure 8:**
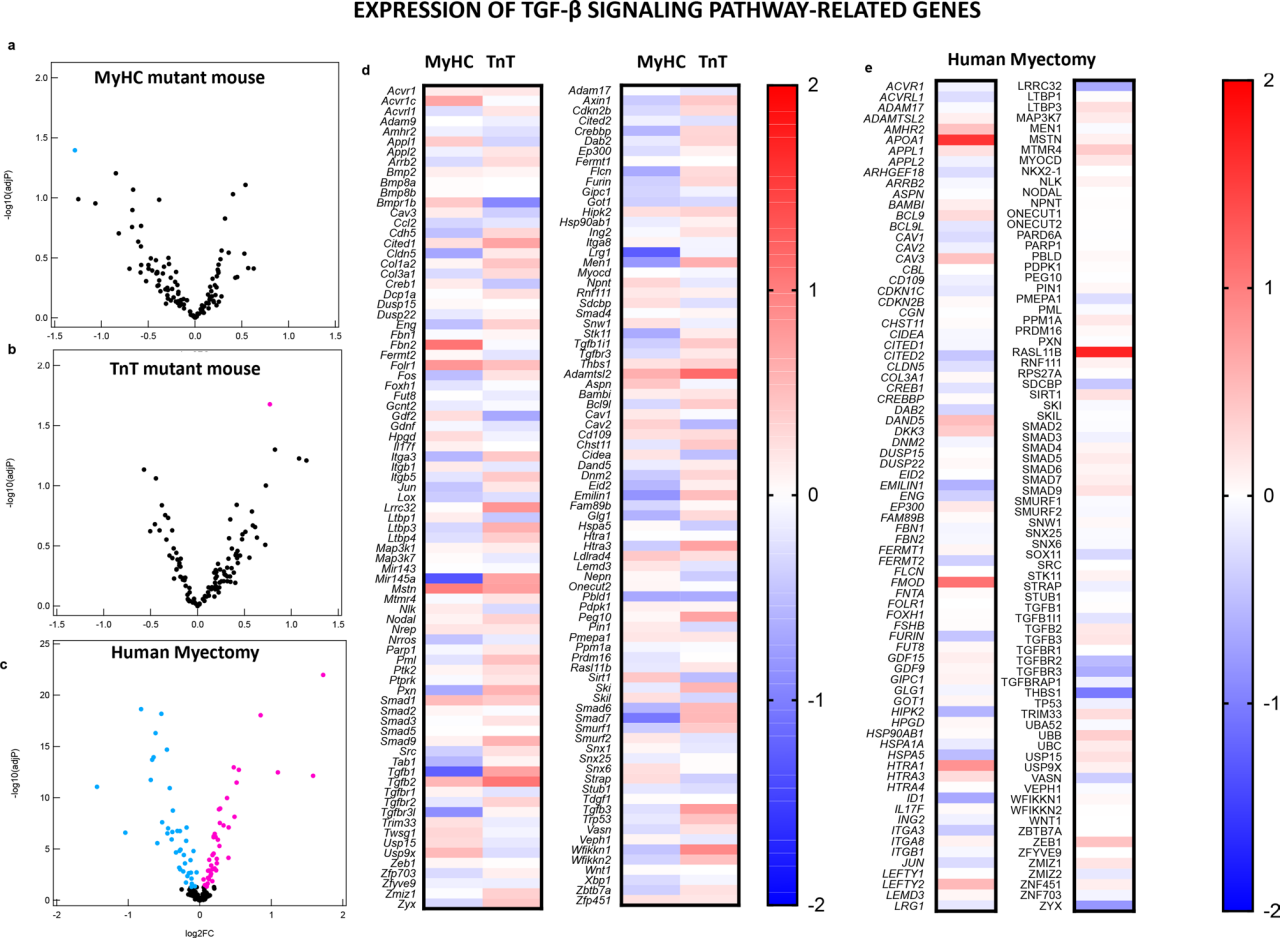
Genes involved in TGF-β signaling pathway and its regulation: Volcano plots of normalized mRNA reads of TGF-β pathway related genes in MyHC-mutant and TnT-mutant mouse hearts (**a**,**b**) and human myectomy tissue (**c**). Heatmap of log2(fold-change) of differentially expressed genes related to TGF-β signaling pathway and its regulators in MyHC-mutant and TnT-mutant mouse hearts (**d**) and in human myectomy tissue (**e**) compared to respective controls. Benjamini–Hochberg method was used to adjust p values for multiple comparisons (n = 3 biological replicates for mouse data; n = 105 HCM patients/n = 39 controls for human data).

Since the TGF-β signaling pathway has been demonstrated to play a major role in generation of cardiac fibrosis, we used gene lists from AmiGO to examine expression of genes involved in the TGF-β signaling pathway and its regulators. At the established disease stage, mutant mice demonstrated significant dysregulation of only one gene each (*Tgfb3, log2FC of 0.77* in TnT-mutants*, Lrg1, log2FC of *− *1.28* in MyHC mutants, Fig. 8a,b,d, Supplemental Table 5), whereas human myectomy tissue demonstrated dysregulation of > 50 genes related to the TGFβ signaling pathway. We observed upregulation of TGFB2, as well as downregulation of TGFBR2, collagen genes, elastin and CTGF in human myectomy tissue*.* (Figs. 7c,e; 8a,e; Supplemental Tables 6–7). The most dysregulated gene in myectomy tissue was *RASL11B*^58^ (log2FC 1.72), which has been associated with pathologic cardiac hypertrophy^58^ in mice and humans.

**Figure 9:**
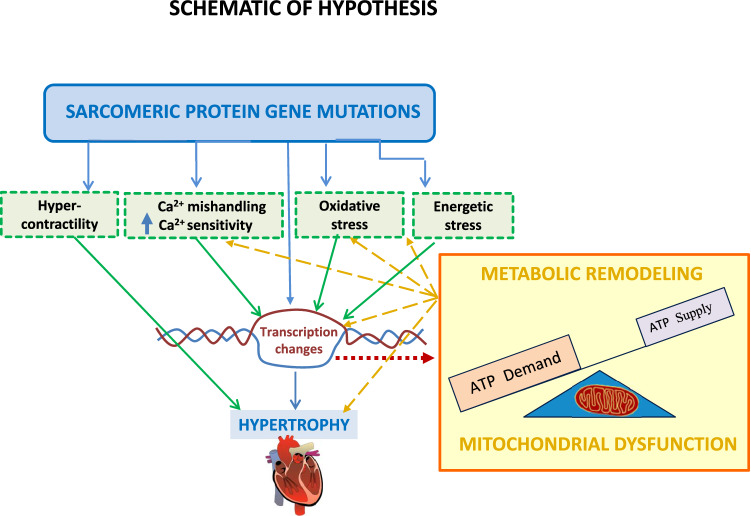
Hypothesis schematic: HCM mutations lead to abnormalities in Ca^2+^ handling, oxidative stress, energetic stress which lead to cardiac transcriptional changes, mitochondrial dysfunction and metabolic gene remodeling. Mitochondrial dysfunction and altered cardiac metabolism contribute to cardiac hypertrophy/fibrosis and exacerbate calcium mishandling, oxidative/energetic stress.

Taken together, our gene expression studies suggest that activation of classic pro-hypertrophic and pro-fibrotic signaling pathways may not be required to maintain hypertrophy and fibrosis at the established disease stage of HCM. Mechanisms that could contribute to maintenance of cardiac hypertrophy and disease progression include mutation-induced cardiac hypercontractility, downregulation of myofilament protein phosphorylation, increased myofilament Ca^2+^ sensitivity and mitochondrial dysfunction^59,60^ (Fig. 9). Our metabolic hypothesis is supported by data from patients diagnosed with mutations in mtDNA or mitochondria-related nuclear genes, who exhibit cardiac hypertrophy^61^. Single cell studies in cardiac myocytes and cardiac fibroblasts would be helpful to examine mechanisms underlying initiation and maintenance of cardiac hypertrophy and fibrosis, induced by sarcomeric protein gene mutations.

### Translational Applications

Our results from human myectomy tissue suggest that cardiac physiology (hemodynamics) is an important driver of gene expression in human HCM. Hence, we anticipate that treatment strategies that address hypercontractility (negative inotropes such as beta blockers, calcium channel blockers, disopyramide and mavacamtem^10^) would be beneficial in obstructive HCM, irrespective of genotype. In contrast, patients with symptomatic, non-obstructive HCM and those with disease progression despite relief of LV obstruction may benefit from therapies tailored to the molecular cardiac phenotype.

### Limitations

Our study has several limitations. We used publicly available human heart microarray datasets, and did not have access to human myectomy tissue for functional studies. The methods used for measuring gene expression in mouse and human HCM were different, namely, microarrays for human data, and sequencing for mouse data. Since mRNA/miRNA was extracted from tissue homogenates we were unable to examine the contribution of individual cell types (cardiac myocytes, fibroblasts, endothelial and vascular smooth muscle cells) to the gene expression changes observed at the established disease stage in mouse-HCM and human-HCM. It would have been interesting to compare gene expression in patients with mutations in *TNNT2* and *MYH7* with corresponding mouse model data. Unfortunately, we did not have access to mutation status of each patient, which precluded direct comparisons of gene expression data stratified by mutation, in mouse and human HCM. However, since genotype-based subgroup analysis in this dataset has revealed few differences in gene expression^12^, we believe that our comparisons between mouse-HCM and human-HCM are valid. An important limitation of our study is the lack of protein expression and metabolic flux studies, to confirm effects of dysregulated gene expression on cardiac metabolism. Since correlations between tissue mRNA and protein expression/function can vary, parallel metabolic flux/metabolomics studies in HCM mouse models and human hearts would be ideal to examine the contribution of abnormalities in cardiac metabolism to the genesis of cardiac HCM phenotype. Advances in hyperpolarized 13C-MRI^62^ provide the opportunity for non-invasive metabolic imaging in human hearts^63^ and experimental models.

## Conclusion

Molecular phenotyping revealed distinct gene expression profiles in two mouse models with non-obstructive HCM at the established disease stage, and in human myectomy tissue from patients with obstructive HCM. We observed a discordance between cardiac hypertrophy detected by echocardiography and activation of pro-hypertrophic signaling pathways and transcription factors (predicted by IPA) in mouse and human HCM. In mouse models, we also identified allele-specific differences in heart size/function, mitochondrial number, Ca^2+^ handling, expression of metabolic pathway genes, as well as similarities, namely, similar amount of interstitial fibrosis, oxidized myocyte redox environment and impairment of ETC complex I function. Our results suggest the need for parallel, longitudinal studies in humans and mouse models expressing homologous mutations, to define the molecular basis of the cardiac phenotype induced by sarcomeric protein gene mutations.

## Supplementary Information

Supplementary Information.
