# Supplementary material for: Differences in molecular phenotype in mouse and human hypertrophic cardiomyopathy
Source: Sci Rep. 2021 Jun 23;11:13163. doi: 10.1038/s41598-021-89451-6 (PMC8222321; doi:10.1038/s41598-021-89451-6)
Supplement: Supplementary file 1 — Supplementary Information. [file 41598_2021_89451_MOESM1_ESM.docx]

**Differences in molecular phenotype in mouse and human hypertrophic cardiomyopathy.**

**SUPPLEMENTARY FIGURES**

**
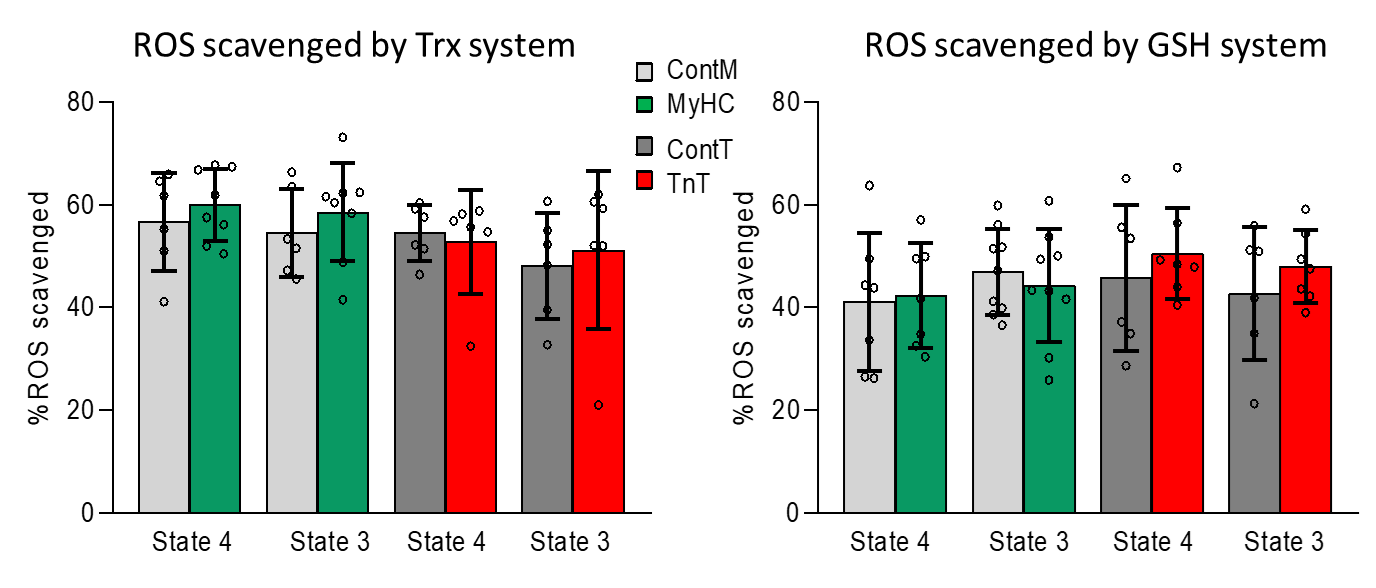
**

Supplemental Figure 1. Mitochondrial reactive oxygen species (ROS) scavenging: MyHC-mutant (MyHC) and TnT-mutant (TnT) mouse mitochondria demonstrated no difference in ROS (H_2_O_2_) scavenging by thioredoxin (Trx) or glutathione(GSH) compared to respective littermate controls (ContM, ContT) during state 4 and state 3 respiration. [n=12 mice for ContM,MyHC,ContT and TnT. Statistical significance was tested using 2-sided unpaired student’s t-test between groups (ContM vs MyHC and ContT vs TnT for each condition)].

Supplemental Figure 2. Ingenuity Pathway Analysis results showing all statistically significant dysregulated pathways in 2 mutant mice (A, B) and human myectomy tissue (C).We used a cutoff value for significance of *p<0.01* and |*Z-score*|>1.Comparison of mRNA data from mutant HCM mice and human myectomy samples with respective controls was performed by ANOVA using the Partek Genomics Suite 7.0 platform.

Supplemental Figure 3. Ingenuity Pathway Analysis results showing all statistically significant dysregulated transcriptional regulators in 2 mutant mice (A, B) and human myectomy tissue (C). We used a cutoff value for significance of *p<0.01* and |*Z-score*|>2.Comparison of mRNA data from mutant HCM mice and human myectomy samples with respective controls was performed by ANOVA using the Partek Genomics Suite 7.0 platform.

Supplemental Figure 4. Ingenuity Pathway Analysis results showing predicted therapies in 2 mutant mice (A, B) and human myectomy tissue (C). We used a cutoff value for significance of *p<0.01* and |*Z-score*|>2.Comparison of mRNA data from mutant HCM mice and human myectomy samples with respective controls was performed by ANOVA using the Partek Genomics Suite 7.0 platform.

Supplemental Figure 5. Differential expression of redox-related genes in human myectomy tissue. Heatmap of log2(fold-change) of normalized mRNA reads and volcano plots of genes involved in reactive oxygen (ROS) generation (A, B) and ROS scavenging (C, D) in human myectomy tissue compared to control hearts. Benjamini-Hochberg method was used to adjust pvalues for multiple comparisons.(n=105 HCM patients, n=39 controls)

**SUPPLEMENTARY TABLES**

| **Supplemental Table 1. Top 20 upregulated and downregulated genes in mouse and human HCM compared to respective controls** | | | | | |
| --- | --- | --- | --- | --- | --- |
| **R403Q-MyHCmouse heart** | | **R92W-TnT mouse heart** | | **Human myectomy tissue** | |
| **Upregulated** | **Downregulated** | **Upregulated** | **Downregulated** | **Upregulated** | **Downregulated** |
| *Casq1* | *Ano8* | *Abhd2* | *A530016L24Rik* | *ACE2* | *AXUD1* |
| *Cep290* | *Arhgef40* | *Arhgef40* | *Aqp4* | *APOA1* | *CD163* |
| *Dhx9* | *Card10* | *Casq1* | *Cenpf* | *C14ORF132* | *CEBPD* |
| *Fam136a* | *Clasrp* | *Cilip* | *Efnb3* | *C21ORF7* | *FCN3* |
| *Far1* | *Fam193b* | *Clasrp* | *Gmnn* | *C7ORF41* | *FKBP5* |
| *Lrrcc1* | *Hma1* | *Egr1* | *Gpr22* | *CA3* | *LYVE1* |
| *Mov10l1* | *Klhdc4* | *Elf4* | *Gpt* | *CENPA* | *MT1M* |
| *Ranbp2* | *Lipe* | *Fat1* | *Il15* | *CLIC6* | *MT1X* |
| *Rasa1* | *Lrg1* | *Fgfr1* | *Mal* | *FRZB* | *MT2A* |
| *Rbm7* | *Lrrc4b* | *Hmha1* | *Mov10l1* | *HS.576694* | *MYH6* |
| *Rnf13* | *Nr2f6* | *Mafg* | *Pfkfb1* | *HSPA2* | *PDK4* |
| *Skap2* | *Pim3* | *Mybpc2* | *Pik3c2a* | *IRX6* | *RASD1* |
| *Suco* | *Ranbp3* | *Slc15a4* | *Ppip5k2* | *LOC100008589* | *S100A8* |
| *Trim12c* | *Rapgef3* | *Slc38a1* | *Pxmp2* | *PIK3IP1* | *S100A9* |
| *Vbp1* | *Smarcd1* | *Slc39a11* | *Rnpc3* | *PROS1* | *SERPINA3* |
| *Yae1d1* | *Snx11* | *Sstr2* | *Sgol2a* | *RASL11B* | *SERPINE1* |
| *Zfp280d* | *Sppl2b* | *Synpo2l* | *Tmx1* | *SCN2B* | *TUBA3C* |
| *Zfp942d* | *Ssbp4* | *Tmc6* | *Tnfaip8* | *SFRP1* | *TUBA3D* |
| *Pcm1* | *Tcf15* | *Tnnt2* | *Zfp942d* | *SMOC2* | *TUBA3E* |
| *1500017e21Rik* | *Zswim4* | *6430548m08Rik* | *4631405J19Rik* | *TPM3* | *ZFP36* |

| **Supplemental Table 2. Complete list of processes obtained by KEGG and GO using significantly dysregulated genes in cardiac tissue obtained by 24 week old mutant mice and human HCM** | | | | | |
| --- | --- | --- | --- | --- | --- |
|  |  |  |  |  |  |
| **R403Q-MyHC mouse heart** | **Pvalue** | **R92W-TnT mouse heart** | **P value** | **Humanmyectomy tissue** | **P value** |
| **KEGG** | | | | | |
| Endocrine and other factor-regulated calcium reabsorption | 0.003 | Valine, leucine and isoleucine degradation | 5.8E-06 | Phagosome | 3.0E-11 |
| Pyruvate metabolism | 0.01 | Propanoate metabolism | 0.001 | Pathogenic Escherichia coli infection | 3.0E-07 |
| Glycerolipid metabolism | 0.02 | Fatty acid degradation | 0.004 | Valine, leucine and isoleucine degradation | 9.0E-07 |
| N-Glycan biosynthesis | 0.03 | Pantothenate and CoA biosynthesis | 0.006 | Staphylococcus aureus infection | 2.0E-06 |
| Regulation of lipolysis in adipocytes | 0.04 | Insulin signaling pathway | 0.01 | Complement and coagulation cascades | 3.0E-06 |
| Protein processing in endoplasmic reticulum | 0.04 | Pyruvate metabolism | 0.02 | Mineral absorption | 2.0.E-05 |
|  |  | Cellular senescence | 0.02 | Viral myocarditis | 3.0E-05 |
|  |  | Huntington disease | 0.03 | Carbon metabolism | 0.0002 |
|  |  | Fatty acid metabolism | 0.03 | Apoptosis | 0.0006 |
|  |  | Phosphatidylinositol signaling system | 0.04 | Propanoate metabolism | 0.0006 |
|  |  | Insulin resistance | 0.04 | HIF-1 signaling pathway | 0.0007 |
|  |  | Tryptophan metabolism | 0.05 | Insulin resistance | 0.001 |
|  |  |  |  | Shigellosis | 0.001 |
|  |  |  |  | Amino sugar and nucleotide sugar metabolism | 0.001 |
|  |  |  |  | Prion diseases | 0.002 |
|  |  |  |  | Salmonella infection | 0.002 |
|  |  |  |  | Pyruvate metabolism | 0.002 |
|  |  |  |  | Arginine and proline metabolism | 0.002 |
| **GO Cellular Component** | | | | | |
| Intrinsic component of organelle membrane | 0.01 | mitochondrial matrix | 3.3E-09 | contractile fiber | 1.8E-09 |
| Polysome | 0.04 | oxidoreductase complex | 4.8E-05 | cell-substrate junction | 5.0E-09 |
|  |  | mitochondrial protein complex | 5.3E-05 | mitochondrial matrix | 3.0E-05 |
|  |  | organelle inner membrane | 0.001 | actin cytoskeleton | 7.0E-05 |
|  |  | mitochondrial membrane part | 0.01 | cell leading edge | 0.0001 |
|  |  | sarcoplasm | 0.02 | blood microparticle | 0.0003 |
|  |  | PML body | 0.02 | outer membrane | 0.0009 |
|  |  | ribosome | 0.02 | pigment granule | 0.001 |
|  |  | contractile fiber | 0.03 | endosomal part | 0.001 |
|  |  | anchored component of membrane | 0.03 | sarcolemma | 0.001 |
|  |  | NADH dehydrogenase complex | 0.03 | cell-cell adherens junction | 0.001 |
|  |  |  |  | ATPase complex | 0.002 |
|  |  |  |  | mitochondrial inner membrane | 0.002 |
| **GO Molecular function** | | | | | |
| ADP binding | 0.006 | coenzyme binding | 0.0002 | cofactor binding | 4.1E-07 |
| single-stranded DNA binding | 0.01 | oxidoreductase activity, acting on the CH-CH group of donors | 0.001 | cell adhesion molecule binding | 1.5E-05 |
| basal transcription machinery binding | 0.02 | electron transfer activity | 0.002 | sulfur compound binding | 1.5E-05 |
| RNA polymerase II transcription factor binding | 0.03 | oxidoreductase activity, acting on the aldehyde or oxo group of donors | 0.003 | structural constituent of cytoskeleton | 1.8E-05 |
| ribonucleoprotein complex binding | 0.03 | sulfur compound binding | 0.004 | oxidoreductase activity, acting on the aldehyde or oxo group of donors | 0.0001 |
| carboxylic ester hydrolase activity | 0.03 | lipase activity | 0.007 | structural constituent of muscle | 0.0001 |
| catalytic activity, acting on DNA | 0.04 | carboxylic ester hydrolase activity | 0.01 | actin binding | 0.0001 |
| beta-catenin binding | 0.04 |  |  | collagen binding | 0.0002 |
| single-stranded RNA binding | 0.04 |  |  | electron carrier activity | 0.0003 |
|  |  |  |  | oxidoreductase activity, acting on the CH-CH group of donors | 0.0005 |
|  |  |  |  | protein binding involved in cell adhesion | 0.0005 |
|  |  |  |  | glycosaminoglycan binding | 0.0006 |
|  |  |  |  | growth factor binding | 0.0008 |
|  |  |  |  | cytokine binding | 0.0009 |
|  |  |  |  | glycoprotein binding | 0.001 |
|  |  |  |  | binding, bridging | 0.001 |
|  |  |  |  | antioxidant activity | 0.001 |
|  |  |  |  | extracellular matrix binding | 0.002 |
|  |  |  |  | GTPase activity | 0.001 |
|  |  |  |  | oxidoreductase activity, acting on a sulfur group of donors | 0.002 |
|  |  |  |  | ATPase binding | 0.003 |
|  |  |  |  | isomerase activity | 0.003 |
| **GO biologic process** | | | | | |
| neutral lipid metabolic process | 0.0004 | fatty acid metabolic process | 2.7E-06 | blood vessel morphogenesis | 2.3E-09 |
| cell redox homeostasis | 0.002 | small molecule catabolic process | 1.2E-05 | negative regulation of growth | 1.6E-08 |
| regulation of binding | 0.005 | lipid modification | 1.7E-05 | response to inorganic substance | 4.4E-08 |
| glycerolipid metabolic process | 0.007 | generation of precursor metabolites and energy | 2.5E-05 | cofactor metabolic process | 4.5E-08 |
| DNA-templated transcription, termination | 0.008 | lipid catabolic process | 0.0002 | heart development | 4.6E-08 |
| morphogenesis of a polarized epithelium | 0.009 | nucleoside bisphosphate metabolic process | 0.0005 | actomyosin structure organization | 8.0E-08 |
| response to temperature stimulus | 0.01 | cellular amino acid metabolic process | 0.002 | cellular metal ion homeostasis | 1.2E-07 |
| actin filament-based movement | 0.01 | organophosphate biosynthetic process | 0.004 | muscle tissue development | 1.6E-07 |
| regulation of organelle assembly | 0.01 | muscle system process | 0.004 | regulation of vasculature development | 1.9E-07 |
| export across plasma membrane | 0.02 | cofactor metabolic process | 0.004 | plasma membrane organization | 2.0E-07 |
| import across plasma membrane | 0.02 | muscle tissue development | 0.005 | cellular response to external stimulus | 3.4E-07 |
| response to ischemia | 0.02 | ribose phosphate metabolic process | 0.005 | cellular response to biotic stimulus | 4.2E-07 |
| lipid homeostasis | 0.02 | fatty acid derivative metabolic process | 0.006 | cell growth | 6.0E-07 |
| regulation of mRNA metabolic process | 0.02 | sulfur compound metabolic process | 0.007 | negative regulation of cell adhesion | 6.8E-07 |
| intraspecies interaction between organisms | 0.02 | response to temperature stimulus | 0.007 | negative regulation of cellular protein localization | 7.6E-07 |
| phospholipid metabolic process | 0.03 | protein-containing complex remodeling | 0.009 | regulation of metal ion transport | 9.8E-07 |
| cell surface receptor signaling pathway involved in cell-cell signaling | 0.03 | purine-containing compound metabolic process | 0.009 | activation of immune response | 1.3E-06 |
| protein localization to cytoskeleton | 0.03 | response to purine-containing compound | 0.01 | regulation of actin filament-based process | 1.7E-06 |
| respiratory gaseous exchange | 0.03 | mitochondrial gene expression | 0.01 | muscle organ development | 1.9E-06 |
| cytoplasmic microtubule organization | 0.03 | glycerolipid metabolic process | 0.02 | regulation of cell activation | 2.1E-06 |
|  |  |  |  | extracellular structure organization | 2.6E-06 |
|  |  |  |  | muscle cell differentiation | 3.0E-06 |
|  |  |  |  | cell redox homeostasis | 3.0E-06 |
|  |  |  |  | response to extracellular stimulus | 3.5E-06 |
|  |  |  |  | fatty acid metabolic process | 3.9E-06 |
|  |  |  |  | negative regulation of cellular component movement | 4.0E-06 |
|  |  |  |  | phagocytosis | 4.4E-06 |
|  |  |  |  | response to oxidative stress | 5.0E-06 |
|  |  |  |  | pyruvate metabolic process | 5.2E-06 |
|  |  |  |  | response to interferon-gamma | 5.5E-06 |
|  |  |  |  | cellular response to organonitrogen compound | 7.2E-06 |
|  |  |  |  | reactive oxygen species metabolic process | 7.3E-06 |
|  |  |  |  | actin filament-based movement | 7.6E-06 |
|  |  |  |  | mesenchyme development | 8.3E-06 |
|  |  |  |  | response to mechanical stimulus | 9.2E-06 |
|  |  |  |  | transition metal ion homeostasis | 9.6E-06 |
|  |  |  |  | negative regulation of locomotion | 1.0E-05 |
|  |  |  |  | membrane raft organization | 1.2E-05 |
|  |  |  |  | actin filament organization | 1.2E-05 |
|  |  |  |  | integrin-mediated signaling pathway | 1.2E-05 |
|  |  |  |  | cellular component assembly involved in morphogenesis | 1.2E-05 |
|  |  |  |  | cellular aldehyde metabolic process | 1.4E-05 |
|  |  |  |  | protein localization to cell periphery | 1.8E-05 |
|  |  |  |  | leukocyte cell-cell adhesion | 2.0E-05 |
|  |  |  |  | endothelium development | 2.0E-05 |
|  |  |  |  | response to drug | 2.2E-05 |
|  |  |  |  | myeloid leukocyte activation | 2.2E-05 |
|  |  |  |  | response to oxygen levels | 2.8E-05 |
|  |  |  |  | pyridine-containing compound metabolic process | 2.9E-05 |
|  |  |  |  | negative regulation of transport | 3.1E-05 |
|  |  |  |  | neuron death | 3.7E-05 |
|  |  |  |  | cell-substrate adhesion | 3.9E-05 |
|  |  |  |  | regulation of protein serine/threonine kinase activity | 4.1E-05 |
|  |  |  |  | response to peptide | 4.5E-05 |
|  |  |  |  | regeneration | 4.8E-05 |
|  |  |  |  | myeloid cell differentiation | 0.0001 |
|  |  |  |  | negative regulation of intracellular signal transduction | 0.0001 |
|  |  |  |  | response to ketone | 0.0001 |
|  |  |  |  | muscle system process | 0.0001 |
|  |  |  |  | membrane biogenesis | 0.0001 |
|  |  |  |  | regulation of response to wounding | 0.0001 |
|  |  |  |  | receptor metabolic process | 0.0001 |
|  |  |  |  | regulation of apoptotic signaling pathway | 0.0001 |
|  |  |  |  | protein activation cascade | 0.0001 |
|  |  |  |  | response to acid chemical | 0.0001 |
|  |  |  |  | connective tissue development | 0.0001 |
|  |  |  |  | autophagy | 0.0001 |
|  |  |  |  | negative regulation of establishment of protein localization | 0.0001 |
|  |  |  |  | negative regulation of phosphorylation | 0.0001 |
|  |  |  |  | regulation of inflammatory response | 0.0001 |
|  |  |  |  | muscle cell proliferation | 0.0001 |
|  |  |  |  | regulation of cytoskeleton organization | 0.0001 |
|  |  |  |  | regulation of lipid metabolic process | 0.0001 |
|  |  |  |  | ameboidal-type cell migration | 0.0002 |
|  |  |  |  | transition metal ion transport | 0.0002 |
|  |  |  |  | in utero embryonic development | 0.0002 |
|  |  |  |  | protein localization to membrane | 0.0002 |
|  |  |  |  | cellular ketone metabolic process | 0.0002 |
|  |  |  |  | regulation of DNA-templated transcription in response to stress | 0.0002 |
|  |  |  |  | homeostasis of number of cells | 0.0002 |
|  |  |  |  | regulation of cell-cell adhesion | 0.0002 |
|  |  |  |  | response to activity | 0.0003 |
|  |  |  |  | response to steroid hormone | 0.0003 |
|  |  |  |  | gland development | 0.0003 |
|  |  |  |  | leukocyte proliferation | 0.0003 |
|  |  |  |  | trabecula morphogenesis | 0.0003 |
|  |  |  |  | regulation of peptidase activity | 0.0004 |
|  |  |  |  | positive regulation of cell adhesion | 0.0004 |
|  |  |  |  | intrinsic apoptotic signaling pathway | 0.0004 |
|  |  |  |  | protein import | 0.0004 |
|  |  |  |  | response to purine-containing compound | 0.0005 |
|  |  |  |  | divalent inorganic cation homeostasis | 0.0005 |
|  |  |  |  | maintenance of location | 0.0006 |
|  |  |  |  | regulation of transporter activity | 0.0007 |
|  |  |  |  | immune response-regulating signaling pathway | 0.0007 |
|  |  |  |  | organic acid biosynthetic process | 0.0007 |
|  |  |  |  | positive regulation of defense response | 0.0008 |
|  |  |  |  | reactive nitrogen species metabolic process | 0.0008 |
|  |  |  |  | response to axon injury | 0.0008 |
|  |  |  |  | ossification | 0.0008 |
|  |  |  |  | negative regulation of proteolysis | 0.0008 |
|  |  |  |  | response to bacterium | 0.0008 |
|  |  |  |  | nuclear transport | 0.0008 |
|  |  |  |  | cellular response to abiotic stimulus | 0.0008 |
|  |  |  |  | lipid homeostasis | 0.0009 |
|  |  |  |  | apoptotic mitochondrial changes | 0.0009 |
|  |  |  |  | negative regulation of immune system process | 0.0009 |
|  |  |  |  | hepaticobiliary system development | 0.001 |
|  |  |  |  | actin cytoskeleton reorganization | 0.001 |
|  |  |  |  | ERK1 and ERK2 cascade | 0.001 |
|  |  |  |  | apoptotic process involved in development | 0.001 |
|  |  |  |  | cellular modified amino acid biosynthetic process | 0.001 |
|  |  |  |  | leukocyte migration | 0.001 |
|  |  |  |  | coagulation | 0.001 |
|  |  |  |  | regulation of homeostatic process | 0.001 |
|  |  |  |  | response to endoplasmic reticulum stress | 0.001 |
|  |  |  |  | negative regulation of response to external stimulus | 0.001 |
|  |  |  |  | ER-nucleus signaling pathway | 0.001 |
|  |  |  |  | multicellular organism metabolic process | 0.001 |
|  |  |  |  | regulation of binding | 0.001 |
|  |  |  |  | sulfur compound metabolic process | 0.001 |
|  |  |  |  | membrane invagination | 0.001 |
|  |  |  |  | response to toxic substance | 0.001 |
|  |  |  |  | cytochrome complex assembly | 0.001 |
|  |  |  |  | positive regulation of cellular component movement | 0.001 |
|  |  |  |  | response to estradiol | 0.001 |
|  |  |  |  | extrinsic apoptotic signaling pathway | 0.002 |
|  |  |  |  | regulation of ATPase activity | 0.002 |
|  |  |  |  | response to transforming growth factor beta | 0.002 |
|  |  |  |  | signal transduction in response to DNA damage | 0.002 |
|  |  |  |  | negative regulation of hydrolase activity | 0.002 |
|  |  |  |  | small molecule catabolic process | 0.002 |
|  |  |  |  | response to arsenic-containing substance | 0.002 |
|  |  |  |  | regulation of intracellular transport | 0.002 |
|  |  |  |  | aging | 0.002 |
|  |  |  |  | leukocyte apoptotic process | 0.002 |
|  |  |  |  | response to fibroblast growth factor | 0.002 |
|  |  |  |  | cell chemotaxis | 0.002 |
|  |  |  |  | regulation of cell morphogenesis | 0.002 |
|  |  |  |  | positive regulation of locomotion | 0.002 |
|  |  |  |  | regulation of transmembrane transport | 0.002 |
|  |  |  |  | positive regulation of cytokine production | 0.003 |
|  |  |  |  | regulation of anatomical structure size | 0.003 |
|  |  |  |  | positive regulation of MAPK cascade | 0.003 |
|  |  |  |  | platelet-derived growth factor receptor signaling pathway | 0.003 |
|  |  |  |  | response to topologically incorrect protein | 0.003 |
|  |  |  |  | fat cell differentiation | 0.004 |
|  |  |  |  | cellular amino acid metabolic process | 0.004 |
|  |  |  |  | heterotypic cell-cell adhesion | 0.004 |
|  |  |  |  | circulatory system process | 0.004 |
|  |  |  |  | protein polymerization | 0.004 |
|  |  |  |  | regulation of sequence-specific DNA binding transcription factor activity | 0.004 |
|  |  |  |  | antigen processing and presentation | 0.004 |
|  |  |  |  | I-kappaB kinase/NF-kappaB signaling | 0.005 |
|  |  |  |  | type 2 immune response | 0.005 |
|  |  |  |  | cell communication by electrical coupling | 0.005 |
|  |  |  |  | osteoblast proliferation | 0.005 |
|  |  |  |  | cell activation involved in immune response | 0.005 |
|  |  |  |  | Notch signaling pathway | 0.005 |
|  |  |  |  | adaptive immune response | 0.005 |
|  |  |  |  | negative regulation of organelle organization | 0.005 |
|  |  |  |  | organ growth | 0.005 |
|  |  |  |  | tissue remodeling | 0.006 |
|  |  |  |  | tissue migration | 0.006 |
|  |  |  |  | exocytosis | 0.007 |
|  |  |  |  | cell junction organization | 0.007 |
|  |  |  |  | tumor necrosis factor superfamily cytokine production | 0.007 |
|  |  |  |  | protein folding | 0.007 |
|  |  |  |  | muscle cell apoptotic process | 0.007 |
|  |  |  |  | muscle cell migration | 0.007 |
|  |  |  |  | divalent inorganic cation transport | 0.007 |
|  |  |  |  | cellular modified amino acid metabolic process | 0.007 |
|  |  |  |  | leukocyte mediated immunity | 0.007 |
|  |  |  |  | protein localization to nucleus | 0.007 |
|  |  |  |  | regulation of cellular response to growth factor stimulus | 0.008 |
|  |  |  |  | negative regulation of transferase activity | 0.008 |
|  |  |  |  | ruffle organization | 0.008 |
|  |  |  |  | response to antibiotic | 0.008 |
|  |  |  |  | organonitrogen compound catabolic process | 0.008 |
|  |  |  |  | regulation of multi-organism process | 0.008 |
|  |  |  |  | macromolecular complex disassembly | 0.008 |
|  |  |  |  | acute inflammatory response | 0.008 |
|  |  |  |  | response to interleukin-1 | 0.009 |
|  |  |  |  | positive regulation of response to external stimulus | 0.01 |
|  |  |  |  | interaction with host | 0.01 |
|  |  |  |  | regulation of body fluid levels | 0.01 |
|  |  |  |  | tube morphogenesis | 0.01 |
|  |  |  |  | mesenchymal cell proliferation | 0.01 |
|  |  |  |  | regulation of mitochondrion organization | 0.01 |
|  |  |  |  | urogenital system development | 0.01 |
|  |  |  |  | multi-multicellular organism process | 0.01 |
|  |  |  |  | respiratory system development | 0.01 |
|  |  |  |  | cell cycle arrest | 0.01 |
|  |  |  |  | mitochondrial membrane organization | 0.01 |
| KEGG; Kyoto Encyclopedia of Genes and Genomes, GO; Gene Ontology. We used a cutoff value for significance of FDR<0.05.  Benjamini-Hochberg method was used to adjust P values for multiple testing. | | | | | |

**Supplemental Table 3. Significantly dysregulated metabolic genes in 24 week old mutant mice (adjusted p<0.05)**

| **R403Q-MyHC mouse heart: DOWNREGULATEDGENES** | | | | **R92W-TnT mouse heart: DOWNREGULATEDGENES** | | |
| --- | --- | --- | --- | --- | --- | --- |
| **Gene** | **Function** | | **Log2FC** | **Gene** | **Function** | **Log2FC** |
| Carnitine O-acetyltransferase (*Crat*) | Mitochochondrial FA transport | | -0.63 | Acetyl-CoA acyltransferase 2 (*Acaa2*) | Beta oxidation | -0.65 |
| Diacylglycerol kinase zeta (*Dgkz*) | Glycerolipid metabolism | | -0.96 | Acyl-CoA dehydrogenase family member 11 (*Acad11*) | Beta oxidation | -0.65 |
| Evolutionarily conserved signaling intermediate in Toll pathway (*Ecsit*) | MAPK signaling pathway | | -0.45 | Short/branched chain acyl-CoA dehydrogenase (*Acadsb*) | FA metabolism | -0.63 |
| Monoglyceride lipase (*Mgll*) | Glycerolipid metabolism | | -0.86 | Acetyl-CoA acetyltransferase (*Acat1*) | FA metabolism | -0.59 |
| Pyruvate kinase (*Pkm*) | Glycolysis/Gluconeogenesis | | -0.45 | Aldehyde dehydrogenase family 6, subfamily A1 (*Aldh6a*1) | Glycerolipid, BCAA/ propionate metabolism | -0.73 |
| Solute carrier family 27 member 1 (*Slc27a1*) | Cellular long chain FA transport | | -1.11 | Branched Chain Amino Acid Transaminase 2 (*Bcat2*) | Glycerolipid, BCAA/ propionate metabolism | -0.59 |
|  | | | | 2-Oxoisovalerate dehydrogenase subunit beta, mitochondrial (*Bckdhb*) | Glycerolipid, BCAA/ propionate metabolism | -0.75 |
|  |  |  |  | Dihydrolipoamide branched chain transacylase E2 (*Dbt*) | Glycerolipid, BCAA/ propionate metabolism | -0.75 |
|  |  |  |  | 2,4-Dienoyl-CoA reductase, mitochondrial (*Decr1*) | Beta oxidation | -0.62 |
|  |  |  |  | Pyruvate dehydrogenase E2 component (*Dlat*) | Pyruvate oxidation | -0.47 |
|  |  |  |  | Extender of the chronological lifespan protein 1 (*Ecl1*) | Chronological cell aging | -0.51 |
|  |  |  |  | Electron transfer flavoprotein subunit alpha (*Etfa*) | Beta-oxidation | -0.55 |
|  |  |  |  | Electron transfer flavoprotein subunit beta (*Etfb*) | Mitochondrial FA oxidation | -0.62 |
|  |  |  |  | Glutaryl-CoA dehydrogenase, mitochondrial (*Gcdh*) | Mitochondrial oxidation of lysine, tryptophan and hydroxylysine | -0.56 |
|  |  |  |  | 3-Hydroxyisobutyrate dehydrogenase  (*Hibadh*) | Glycerolipid, BCAA/ propionate metabolism | -0.53 |
|  |  |  |  | Lipoprotein lipase (*Lpl*) | Cholesterol metabolism | -0.58 |
|  |  |  |  | Methylcrotonoyl-Coenzyme A carboxylase 2  (*Mccc2*) | Glycerolipid, BCAA/ propionate metabolism | -0.70 |
|  |  |  |  | NADH dehydrogenase 1 alpha subcomplex subunit 10 (*Ndufa10*) | OxPhos | -0.48 |
|  |  |  |  | NADH dehydrogenase 1 beta subcomplex subunit 8 (*Ndufb8*) | OxPhos | -0.71 |
|  |  |  |  | NADH dehydrogenase Fe-S protein 2 (*Ndufs2*) | OxPhos | -0.54 |
|  |  |  |  | Pyruvate dehydrogenase E1 subunit alpha 1 (*Pdha1*) | Pyruvate oxidation | -0.45 |
|  |  |  |  | Succinate-CoA ligase GDP/ADP-forming subunit alpha (*Sucla2*) | Krebs cycle | -0.71 |
|  |  |  |  | Ubiquinol-cytochrome c reductase iron-sulfur subunit (*Uqcrfs1*) | OxPhos | -0.61 |
|  | |  | | | |  |

**Supplemental Table 4. Significantly dysregulated genes in human myectomy tissue (adjusted p<0.05)**

| **DOWNREGULATED GENES** | | | | **UPREGULATED GENES** | | |
| --- | --- | --- | --- | --- | --- | --- |
| **Gene** | **Function** | | **Log2FC** | **Gene** | **Function** | **Log2FC** |
| Acetoacetyl-CoA synthetase (*AACS*) | Glycerolipid, BCAA/propionate metabolism | | -0.22 | 4-Aminobutyrate aminotransferase, mitochondrial (ABAT) | Glycerolipid, BCAA/ propionate metabolism | 0.14 |
| ATP binding cassette subfamily D member 3 (*ABCD3*) | FA transporter/Peroxisome biogenesis | | -0.13 | Isobutyryl-CoA dehydrogenase, mitochondrial (*ACAD8*) |  | 0.35 |
| Acetyl-CoA carboxylase beta (*ACACB*) | Glycerolipid, BCAA/propionate metabolism | | -0.27 | Acyl-CoA dehydrogenase family member 11 *(ACAD11)* | Beta oxidation | 0.30 |
| Very long chain acyl-CoA dehydrogenase *(ACADVL)* | Beta oxidation | | -0.25 | Acyl-CoA dehydrogenase long chain *(ACADL)* |  | 0.28 |
| Acetyl-CoA acetyltransferase 2 *(ACAT2)* | [Val, Leu, Ile degradation](http://pathcards.genecards.org/card/valine_leucine_and_isoleucine_degradation), [carbon metabolism](http://pathcards.genecards.org/card/carbon_metabolism) | | -0.12 | Acyl-CoA dehydrogenase medium chain *(ACADM)* |  | 0.46 |
| ATP citrate lyase *(ACLY)* | Lipid biosynthesis and metabolism | | -0.32 | Short/branched chain acyl-CoA dehydrogenase *(ACADSB)* | FA metabolism | 0.40 |
| Acyl-CoA oxidase *(ACOX3)* | FA metabolism | | -0.09 | Acetyl-CoA acetyltransferase *(ACAT1)* |  | 0.34 |
| Acyl-CoA synthetase long chain family member 5 *(ACSL5)* | Beta oxidation | | -0.18 | Aconitase 1 *(ACO1)* | Krebs cycle | 0.14 |
| Alcohol dehydrogenase 1A *(ADH1A)* | Signaling by retinoic acid and drug metabolism | | -0.62 | Acyl-CoA oxidase 1 *(ACOX1)* | Beta oxidation | 0.21 |
| Alcohol dehydrogenase 1B *(ADH1B)* |  | | -0.28 | Acetyl-coenzyme A synthetase 2-like, mitochondrial (ACSS1) | Glycerolipid, BCAA/ propionate metabolism | 0.26 |
| Alcohol dehydrogenase 1C *(ADH1C)* |  | | -0.16 | Alcohol dehydrogenase 5 *(ADH5)* | Drug metabolism | 0.42 |
| Lysophosphatidylinositol acyltransferase *(AGPAT3)* | Triacylglycerol biosynthesis | | -0.06 | Acylglycerol kinase *(AGK)* | Glycerolipid, BCAA/ propionate metabolism | 0.20 |
| Aldo-keto reductase family 1 member A1 *(AKR1A1)* | Naphthalene metabolism | | -0.20 | Glycogen debranching enzyme *(AGL)* | Glycogen degradation | 0.30 |
| Aldo-keto reductase family 1 member B1 *(AKR1B1)* |  | | -0.18 | Lysophosphatidate acyltransferase *(AGPAT1)* | Lipid biosynthesis and signal transduction | 0.19 |
| Aldehyde dehydrogenase *(ALDH1A3)* | Signaling by retinoic acid and drug metabolism | | -0.75 | Lysophosphatidate acyltransferase *(AGPAT2)* |  | 0.13 |
| Fructose-biphosphate aldolase, class I *(ALDOA)* | Glycolysis/Gluconeogenesis | | -0.21 | Methylmalonate-semialdehyde dehydrogenase [acylating], mitochondrial (ALDH6A1) | Glycerolipid, BCAA/ propionate metabolism | 0.45 |
| Aldehyde oxidase 1 (AOX1) | Glycerolipid, BCAA/propionate metabolism | | -0.31 | Aldehyde dehydrogenase 2 family member *(ALDH2)* | Alcohol metabolism | 0.16 |
| ATP synthase membrane subunit c locus 1 *(ATP5G1)* | OxPhos | | -0.21 | Aldehyde dehydrogenase 3 family member A2 *(ALDH3A2)* | Sphingolipid metabolisms | 0.13 |
| ATP H^+^ transporting V0 subunit a1 *(ATP6V0A1)* | Acidification of eukaryotic intracellular organelles | | -0.37 | Aldehyde dehydrogenase 3 family member B1 *(ALDH3B1)* |  | 0.09 |
| ATP H^+^ transporting V0 subunit a2 *(ATP6V0A2)* |  | | -0.15 | Aldehyde dehydrogenase family 7 member A1 *(ALDH7A1)* | Val, Leu, Ile degradation | 0.34 |
| V-type H^+^ transporting V0 subunit c *(ATP6V0C)* |  | | -0.41 | Aldolase, fructose-biphosphate C *(ALDOC)* | Glycolysis/Gluconeogenesis | 0.27 |
| ATPase H^+^ transporting V1 subunit A *(ATP6V1A)* |  | | -0.22 | ATP synthase F1 subunit epsilon *(ATP5F1)* | OxPhos | 0.13 |
| ATPase H^+^ transporting V1 subunit F *(ATP6V1F)* |  | | -0.54 | ATP synthase membrane subunit c locus 3 *(ATP5G3)* |  | 0.26 |
| Branched chain amino acid transaminase 1, cytosolic *(BCAT1*) | Glycerolipid, BCAA/propionate metabolism | | -0.47 | ATP synthase membrane subunit G *(ATP5L)* |  | 0.26 |
| Branched chain amino acid transaminase 2 (*BCAT2*) |  | | -0.15 | ATPase H^+^ transporting V1 subunit D *(ATP6V1D)* | Acidification of eukaryotic intracellular organelles | 0.25 |
| Carboxyl ester lipase *(CEL)* | Lipoprotein metabolism | | -0.63 | ATPase H^+^ transporting V1 subunit E1 *(ATP6V1E1)* |  | 0.16 |
| Cytochrome c1 *(CYC1)* | OxPhos | | -0.14 | ATPase H^+^ transporting V1 subunit G2 *(ATP6V1G2)* |  | 0.30 |
| Diacylglycerol O-acyltransferase 1 *(DGAT1)* | FA metabolism | | -0.22 | ATPase H^+^ transporting V1 subunit H *(ATP6V1H)* |  | 0.16 |
| Diacylglycerol kinase alpha *(DGKA)* | Signaling by GPCR and RET | | -0.06 | ATP synthase mitochondrial F1 complex assembly factor 1 *(ATPAF1)* | OxPhos | 0.46 |
| Enolase 1 *(ENO1)* | Glycolysis/Gluconeogenesis | | -0.82 | ATP synthase mitochondrial F1 complex assembly factor 2 *(ATPAF2)* |  | 0.28 |
| Electron transfer flavoprotein subunit beta *(ETFB)* | Beta oxidation | | -0.18 | ATPase inhibitor, mitochondrial *(ATPIF1)* |  | 0.79 |
| Fructose-biphosphatase 1 *(FBP1)* | Glycolysis/Gluconeogenesis | | -0.55 | AU RNA binding methylglutaconyl- CoA hydratase *(AUH)* | Val, Leu, Ile degradation | 0.30 |
| Glucose-6-phosphatase catalytic subunit 3 *(G6PC3)* |  | | -0.20 | 2-oxoisovalerate dehydrogenase subunit alpha, mitochondrial (BCKDHA) | Glycerolipid, BCAA/propionate metabolism | 0.25 |
| Glycerol kinase *(GK)* | Beta oxidation, triacylglycerol biosynthesis | | -0.11 | CD 36 molecule *(CD36)* | [Binding and Uptake of Ligands by Scavenger Receptors](http://pathcards.genecards.org/card/binding_and_uptake_of_ligands_by_scavenger_receptors) | 0.26 |
| Galactosidase alpha *(GLA)* | Sphingolipid metabolism | | -0.51 | Cytochrome c oxidase assembly protein subunit 15 *(COX15)* | OxPhos | 0.30 |
| Glycerate kinase *(GLYCTK)* | Fructose metabolism | | -0.11 | Cytochrome c oxidase assembly protein subunit 17 *(COX17)* |  | 0.20 |
| Hexokinase 1 *(HK1)* | Glycolysis | | -0.18 | Cytochrome c oxidase subunit 7A2 *(COX7A2)* |  | 0.15 |
| Hexokinase 2 *(HK2)* |  | | -0.35 | Carnitine palmitoyltransferase 1B *(CPT1B)* | Beta oxidation | 0.75 |
| Hexokinase 3 *(HK3)* |  | | -0.46 | Carnitine palmitoyltransferase 2 *(CPT2)* |  | 0.50 |
| Hydroxysteroid 17-beta dehydrogenase 10 *(HSD17B10)* | Val, Leu, Ile degradation, beta oxidation | | -0.25 | Lipoamide acyltransferase component of branched-chain alpha-keto acid dehydrogenase complex, mitochondrial (*DBT*) | Glycerolipid, BCAA/propionate metabolism | 0.25 |
| Isocitrate dehydrogenase (NADP^+^) 1 *(IDH1)* |  | | -0.16 | Diacylglycerol kinase iota *(DGKI)* | Signaling by GPCR and RET | 0.09 |
| Isocitrate dehydrogenase (NADP^+^) 2 *(IDH2)* |  | | -0.31 | Pyruvate dehydrogenase E2 component *(DLAT)* | Pyruvate oxidation | 0.26 |
| Isocitrate dehydrogenase (NAD^+^) 3 non-catalytic subunit gamma *(IDH3G)* |  | | -0.14 | Dihydrolipoamide dehydrogenase *(DLD)* | Pyruvate oxidation,Val, Leu, Ile degradation | 0.42 |
| Lactate dehydrogenase A *(LDHA)* | Glycolysis | | -0.94 | Ethylmalonyl-CoA decarboxylase  (*ECHDC1*) | Glycerolipid and branched chain AA/propionate metabolism | 0.18 |
| Lipase G, endothelial type*(LIPG)* | Lipoprotein metabolism | | -0.64 | Enoyl-CoA hydratase, short chain 1 *(ECHS1)* | Val, Leu, Ile degradation, beta oxidation | 0.17 |
| Malate dehydrogenase 2 *(MDH2)* | Krebs cycle | | -0.12 | Enoyl-CoA hydratase and 3-hydroxyacyl CoA dehydrogenase *(EHHADH)* |  | 0.26 |
| NADH dehydrogenase 1 alpha subcomplex subunit 4 *(NDUFA4L2)* | OxPhos | | -0.67 | Enolase 2 *(ENO2)* | Glycolysis/Gluconeogenesis | 0.58 |
| NADH dehydrogenase 1 alpha subcomplex assembly factor 2 *(NDUFAF2)* | Thermogenesis | | -0.17 | Electron transfer flavoprotein subunit alpha *(ETFA)* | Beta oxidation | 0.22 |
| NADH dehydrogenase flavoprotein 1 *(NDUFV1)* | OxPhos | | -0.21 | Fatty acid-binding protein 3, muscle and heart *(FABP3)* | FA metabolism | 0.38 |
| Succinyl-CoA:3-ketoacid coenzyme A transferase 2, mitochondrial (*OXCT2*) | Glycerolipid, BCAA/propionate metabolism | | -0.37 | Galactose mutarotase*(GALM)* | Galactose metabolism | 0.29 |
| Phosphoenolpyruvate carboxykinase 2, mitochondrial *(PCK2)* | Glycolysis/Gluconeogenesis | | -0.24 | Glycerol-3-phosphae acyltransferase, mitochondrial *(GPAM)* | Glycerolipid, BCAA/propionate metabolism | 0.43 |
| Inorganic pyrophosphatase 1 *(PPA1)* | Phosphate metabolism | | -0.24 | Glucose-6-phosphate isomerase *(GPI)* | Glycolysis/Cori cycle | 0.17 |
| Glycogen phosphorylase L *(PYGL)* | Galactose metabolism | | -0.38 | Glyoxylate and hydroxypyruvate reductase *(GRHPR)* | metabolism | 0.22 |
| Solute carrier family 2 member 1 *(SLC2A1)* | Hexose transport and Cori Cycle | | -0.77 | Glycogen synthase 1 *(GYS1)* | Glycogen metabolism | 0.50 |
| Solute carrier family 25 member 44 (*SLC25A44*) | Glycerolipid, BCAA/propionate metabolism | | -0.28 | Hydroxyacyl-CoA dehydrogenase *(HADH)* | Val, Leu, Ile degradation, beta oxidation | 0.41 |
| T cell immune regulator 1, ATPase H+ transporting V0 subunit a3 *(TCIRG1)* | Proton-transporting ATPase activity | | -0.27 | 3-hydroxyisobutyryl-CoA hydrolase, mitochondrial (*HIBCH*) | Glycerolipid, BCAA/propionate metabolism | 0.31 |
| UDP-glucose pyrophorylase 2 *(UGP2)* | Galactose metabolism | | -0.17 | Hydroxymethylglutaryl-CoA lyase, mitochondrial (HMGCL) |  | 0.32 |
|  | | | | Hydroxysteroid 17-beta dehydrogenase 4 *(HSD17B4)* | Peroxisomal beta-oxidation | 0.15 |
|  |  |  |  | Hydroxysteroid 17-beta dehydrogenase 8 *(HSD17B8)* | Fatty acid biosynthesis | 0.22 |
|  |  |  |  | Isovaleryl-CoA dehydrogenase *(IVD)* | Val, Leu, Ile degradation | 0.33 |
|  |  |  |  | Lactate dehydrogenase B *(LDHB)* | Glycolysis | 0.20 |
|  |  |  |  | Methylcrotonoyl-CoA carboxylase subunit alpha, mitochondrial (*MCCC1*) | Glycerolipid, BCAA/propionate metabolism | 0.29 |
|  |  |  |  | Methylmalonyl-CoA epimerase, mitochondrial (*MCEE*) |  | 0.18 |
|  |  |  |  | Malate dehydrogenase 1 (*MDH1*) | Krebs cycle | 0.13 |
|  |  |  |  | Malonyl-CoA decarboxylase, mitochondrial (*MLYCD*) | Glycerolipid, BCAA/propionate metabolism | 0.28 |
|  |  |  |  | NADH dehydrogenase 1 alpha subcomplex subunit 8 *(NDUFA8)* | OxPhos | 0.27 |
|  |  |  |  | NADH dehydrogenase 1 alpha subcomplex subunit 9 *(NDUFA9)* |  | 0.16 |
|  |  |  |  | NADH dehydrogenase 1 beta subcomplex subunit 4 *(NDUFB4)* |  | 0.09 |
|  |  |  |  | NADH dehydrogenase 1 beta subcomplex subunit 5 *(NDUFB5)* |  | 0.29 |
|  |  |  |  | NADH dehydrogenase 1 beta subcomplex subunit 7 *(NDUFB7)* |  | 0.18 |
|  |  |  |  | NADH dehydrogenase 1 subunit C1 *(NDUFC1)* |  | 0.16 |
|  |  |  |  | NADH dehydrogenase 1 subunit C2 *(NDUFC2)* |  | 0.13 |
|  |  |  |  | NADH dehydrogenase Fe-S protein 1 *(NDUFS1)* |  | 0.25 |
|  |  |  |  | NADH dehydrogenase Fe-S protein 2 *(NDUFS2)* |  | 0.26 |
|  |  |  |  | NADH dehydrogenase Fe-S protein 3 *(NDUFS3)* |  | 0.12 |
|  |  |  |  | NADH dehydrogenase flavoprotein 3 *(NDUFV3)* |  | 0.11 |
|  |  |  |  | Oxoglutarate dehydrogenase like *(OGDHL)* | Krebs cycle | 0.47 |
|  |  |  |  | 3-oxoacid CoA-transferase 1 *(OXCT1)* | Val, Leu, Ile degradation, ketone body metabolism | 0.64 |
|  |  |  |  | Propionyl-CoA carboxylase beta chain, mitochondrial (*PCCB*) | Glycerolipid, BCAA/propionate metabolism | 0.39 |
|  |  |  |  | Pyruvate dehydrogenase E1 subunit beta (*PDHB*) | Pyruvate oxidation | 0.28 |
|  |  |  |  | Enoyl-CoA delta isomerase 2 (*PECI*) | Beta oxidation | 0.27 |
|  |  |  |  | Phosphofructokinase, muscle (*PFKM*) | Glycolysis/Gluconeogenesis | 0.34 |
|  |  |  |  | Phytanoyl-CoA 2-hydroxylase (*PHYH*) | Peroxisome function | 0.42 |
|  |  |  |  | Glycogen phosphorylase B (*PYGB*) | Glycogen degradation | 0.48 |
|  |  |  |  | Succinate dehydrogenase complex flavoprotein subunit A (*SDHA*) | Krebs cycle | 0.23 |
|  |  |  |  | Succinate dehydrogenase complex subunit D (*SDHD*) |  |  |
|  |  |  |  | Serine dehydratase-like (*SDSL*) | Glycerolipid, BCAA/propionate metabolism | 0.78 |
|  |  |  |  | Solute carrier family 25 member 11 *(SLC25A11)* | Glycolysis/Gluconeogenesis | 0.46 |
|  |  |  |  | Solute carrier family 25 member 20 *(SLC25A20)* | Carnitine shuttle | 0.28 |
|  |  |  |  | Solute carrier family 27 member 1 *(SLC27A1)* | Cellular long chain FA transport | 0.31 |
|  |  |  |  | Succinate-CoA ligase ADP-forming subunit beta *(SUCLA2)* | Krebs cycle | 0.28 |
|  |  |  |  | Succinate-CoA ligase GDP/ADP-forming subunit alpha *(SUCLG1)* |  | 0.13 |
|  |  |  |  | Ubiquinol-cytochrome c reductase core protein 2 *(UQCRC2)* | OxPhos | 0.20 |
|  |  |  |  | Ubiquinol-cytochrome c reductase complex III subunit VII *(UQCRQ)* |  | 0.21 |
|  | | FA: fatty acid, OxPhos: oxidative phosphorylation | | | |  |

**Supplemental Table 5: Significantly dysregulated (adjusted P<0.05) genes involved in redox, cardiac hypertrophy and TGF-beta signaling, in HCM mutant mice.**

|  | **R403Q-MyHC mouse heart** | | **R92W-TnT mouse heart** | |
| --- | --- | --- | --- | --- |
| **Gene** | **Log2(Fold-Change)** | **Adjusted**  **pvalue** | **Log2(Fold-Change)** | **Adjusted**  **pvalue** |
| **ROS generation and ROS scavenging** | | | | |
| *Cygb* | -0.22 | 0.587 | 0.70 | 0.031 |
| *Gstm7* | 0.15 | 0.711 | -0.69 | 0.037 |
| *Sod2* | -0.13 | 0.627 | -0.47 | 0.046 |
| **Cardiac Hypertrophy and Regulators** | | | | |
| *Rock1* | 0.12 | 0.697 | -0.55 | 0.025 |
| *Slc25a4* | -0.13 | 0.713 | -0.64 | 0.018 |
| *Trip10* | -0.73 | 0.048 | 0.06 | 0.893 |
| **TGF beta signaling pathway and Regulators** | | | | |
| *Lrg1* | -1.28 | 0.040 | -0.10 | 0.889 |
| *Tgfb3* | -0.24 | 0.574 | 0.77 | 0.021 |

Benjamini-Hochberg method was used to adjust pvalues for multiple comparisons (n=3 biological replicates).

**Supplemental Table 6: Significantly dysregulated (adjusted *p<0.05*) genes implicated in ROS generation/scavenging in human myectomy tissue.**

| **ROS generation** | | | **ROS scavenging** | | |
| --- | --- | --- | --- | --- | --- |
| **Gene** | **Log2(Fold-Change)** | **Adjusted**  **pvalue** | **Gene** | **Log2(Fold-Change)** | **Adjusted**  **pvalue** |
| *AATF* | -0.23 | 0.0007 | *ALOX5AP* | -1.39 | 7.9E-25 |
| *ABCG2* | 0.20 | 6.4E-09 | *APOE* | 0.61 | 5.6E-05 |
| *ACP5* | -0.07 | 0.04 | *APOM* | 0.19 | 2.3E-07 |
| *ADA* | -0.31 | 8.8E-07 | *APP* | 0.81 | 5.9E-10 |
| *AGT* | -0.25 | 0.0001 | *CAT* | 0.41 | 6.8E-09 |
| *ARG2* | -0.46 | 5.9E-06 | *CCS* | -0.11 | 0.01 |
| *CLEC7A* | -0.07 | 0.0004 | *CD36* | 0.26 | 0.003 |
| *CTSS* | -0.28 | 5.5E-11 | *CDKN1B* | 0.45 | 8.5E-11 |
| *CYBA* | -0.98 | 1.5E-25 | *CYGB* | -0.37 | 3.1E-07 |
| *CYBB* | -0.77 | 3.3E-19 | *GLRX* | 0.43 | 0.002 |
| *DMD* | 0.07 | 0.02 | *GPX1* | -0.36 | 1.6E-09 |
| *EDN1* | -0.35 | 2.5E-05 | *GPX3* | -0.18 | 0.01 |
| *FPR2* | -0.14 | 5.1E-08 | *GSTK1* | 0.20 | 0.0009 |
| *FYN* | 0.12 | 0.0002 | *HBZ* | -0.07 | 0.02 |
| *GNAI2* | -0.26 | 4.5E-06 | *HP* | -0.10 | 0.003 |
| *GNAI3* | -0.14 | 0.0131 | *LTC4S* | 0.14 | 0.008 |
| *GSTP1* | -0.17 | 0.0002 | *MGST1* | -0.68 | 9.0E-27 |
| *HIF1A* | -0.31 | 5.6E-07 | *MGST2* | 0.35 | 1E-08 |
| *ITGAM* | -0.57 | 6.5E-21 | *MGST3* | 0.46 | 3.0E-16 |
| *ITGB2* | -1.41 | 1.5E-27 | *MSRB2* | 0.21 | 2.2E-05 |
| *MAOB* | 0.44 | 1.0E-06 | *MT3* | -0.40 | 3.0E-07 |
| *MOCOS* | -0.12 | 0.0008 | *NQO1* | -0.20 | 0.0005 |
| *NCF1* | -0.11 | 0.002 | *NXN* | -0.18 | 0.0004 |
| *NLRP3* | -0.25 | 4.2E-15 | *PARK7* | 0.16 | 0.0006 |
| *NOS3* | -0.49 | 5.4E-07 | *PRDX1* | -0.27 | 9.6E-09 |
| *PRKCD* | -0.73 | 7.9E-35 | *PRDX3* | 0.33 | 0.0002 |
| *RIPK3* | -0.11 | 0.003 | *PRDX4* | -0.31 | 3.8E-08 |
| *SOD1* | 0.15 | 0.008 | *PRDX6* | -0.46 | 9.4E-15 |
| *SOD2* | -0.24 | 0.0002 | *PTGDS* | -0.42 | 1.8E-05 |
| *STAT3* | -1.12 | 2.3E-28 | *PTGS1* | -0.15 | 0.0003 |
| *SYK* | -0.44 | 2.1E-14 | *PTGS2* | -0.14 | 0.006 |
| *TYROBP* | -1.14 | 3.1E-21 | *PXDN* | -0.32 | 0.02 |
|  |  |  | *PXDNL* | 0.59 | 5.0E-09 |
|  |  |  | *RGN* | 0.18 | 0.004 |
|  |  |  | *S100A9* | -2.91 | 8.2E-37 |
|  |  |  | *SMOX* | -0.05 | 0.02 |
|  |  |  | *SNCA* | 1.01 | 8.6E-15 |
|  |  |  | *SRXN1* | -0.29 | 0.0004 |
|  |  |  | *TP53INP1* | 0.54 | 9.6E-14 |
|  |  |  | *TPO* | -0.13 | 0.0001 |
|  |  |  | *TXN* | -0.25 | 0.0002 |
|  |  |  | *TXNRD1* | -0.26 | 0.02 |
|  |  |  | *UBIAD1* | -0.29 | 6.6E-06 |

Benjamini-Hochberg method was used to adjust pvalues for multiple comparisons.(n= 105 HCM patients, n = 39 controls). Genes with log2FC >1 or <-1 are indicated by grey shading.

**Supplemental Table 7: Significantly dysregulated (adjusted *p<0.05*) genes implicated in cardiac hypertrophy in human myectomy tissue.**

| **Gene** | **Log_2_(Fold-Change)** | **Adjusted**  **pvalue** |
| --- | --- | --- |
| *AGT* | -0.25 | 0.0001 |
| *ATP2A2* | -0.76 | 3.7E-16 |
| *CAV3* | 0.47 | 1.6E-13 |
| *CTDP1* | -0.07 | 0.008 |
| *EDN1* | -0.35 | 2.5E-05 |
| *ERRFI1* | -0.77 | 9.2E-21 |
| *EZH2* | -0.05 | 0.03 |
| *FOXO1* | -0.11 | 0.02 |
| *G6PD* | -0.34 | 4.2E-12 |
| *GATA4* | 0.23 | 0.0001 |
| *GATA5* | 0.18 | 0.03 |
| *GATA6* | 0.18 | 6.7E-05 |
| *GSK3A* | 0.10 | 0.01 |
| *HAMP* | -1.02 | 1.3E-09 |
| *HAND2* | 0.26 | 5.5E-05 |
| *HDAC2* | 0.23 | 4.6E-05 |
| *HDAC4* | 0.19 | 0.03 |
| *HEY2* | -0.93 | 1.8E-08 |
| *HTR2B* | 0.54 | 1.4E-10 |
| *KLF15* | -0.33 | 2.0E-06 |
| *LEP* | -0.19 | 0.04 |
| *LMCD1* | -1.43 | 2.3E-15 |
| *LMNA* | -0.51 | 1.0E-16 |
| *MEF2C* | 0.29 | 0.0001 |
| *MYH6* | -1.91 | 8.8E-37 |
| *NOTCH1* | -0.31 | 7.7E-06 |
| *NPPA* | 0.93 | 0.03 |
| *P2RX4* | -0.15 | 0.002 |
| *PAK1* | 0.38 | 6.6E-08 |
| *PDE5A* | 0.77 | 1.9E-22 |
| *PDLIM5* | 0.38 | 9.0E-06 |
| *PPARA* | 0.09 | 0.02 |
| *RGS2* | -0.80 | 1.6E-12 |
| *RGS4* | 1.22 | 4.2E-21 |
| *ROCK1* | 0.16 | 0.001 |
| *RYR2* | 0.87 | 1.0E-11 |
| *SLC9A1* | -0.42 | 1.0E-07 |
| *SORBS2* | 0.83 | 5.4E-29 |
| *TCAP* | -0.40 | 0.0002 |
| *TIAM1* | -0.08 | 0.03 |
| *TNFRSF1A* | -0.75 | 8.0E-20 |
| *TNFRSF1B* | -0.57 | 1.4E-16 |

Benjamini-Hochberg method was used to adjust pvalues for multiple comparisons.(n= 105 HCM patients, n = 39 controls). Genes with log2FC >1 or <-1 are indicated by grey shading.

**Supplemental Table 8: Significantly dysregulated (adjusted *p<0.05*) genes involved in the TGF-beta signaling pathway and its regulation in human myectomy tissue.**

| **Gene** | **Log_2_(Fold-Change)** | **Adjusted**  **P value** |
| --- | --- | --- |
| *ACVRL1* | -0.27 | 1.1E-05 |
| *AMHR2* | 0.49 | 7.2E-09 |
| *APOA1* | 1.58 | 7.2E-13 |
| *APPL1* | 0.23 | 0.001 |
| *APPL2* | -0.12 | 0.04 |
| *ARHGEF18* | -0.28 | 1.7E-07 |
| *ARRB2* | -0.07 | 0.04 |
| *BCL9* | 0.28 | 1.1E-09 |
| *BCL9L* | -0.21 | 0.0002 |
| *CAV1* | -0.28 | 0.00001 |
| *CAV3* | 0.47 | 1.6E-13 |
| *CD109* | -0.12 | 0.002 |
| *CDKN1C* | -0.19 | 0.01 |
| *CITED2* | -0.45 | 2.9E-07 |
| *CREB1* | -0.23 | 0.001 |
| *DAB2* | -0.33 | 2.1E-05 |
| *DAND5* | 0.51 | 3.3E-12 |
| *DKK3* | 0.40 | 7.2E-05 |
| *EMILIN1* | -0.62 | 5E-17 |
| *ENG* | -0.38 | 2.1E-07 |
| *EP300* | 0.18 | 3.9E-05 |
| *FERMT2* | -0.38 | 1.7E-09 |
| *FMOD* | 1.09 | 3.3E-13 |
| *FURIN* | -0.46 | 2.0E-15 |
| *GDF15* | 0.13 | 0.001 |
| *GDF9* | 0.07 | 0.03 |
| *GIPC1* | 0.10 | 0.04 |
| *HIPK2* | -0.59 | 2.7E-06 |
| *HSPA5* | -0.53 | 2.4E-08 |
| *HTRA1* | 0.85 | 8.8E-19 |
| *HTRA3* | 0.28 | 2.8E-08 |
| *ID1* | -0.69 | 1.8E-12 |
| *ING2* | -0.11 | 0.02 |
| *ITGA3* | -0.42 | 1.1E-11 |
| *JUN* | -0.29 | 0.0006 |
| *LEFTY2* | 0.55 | 1.8E-13 |
| *LRG1* | -0.19 | 1.5E-06 |
| *LRRC32* | -0.67 | 1.9E-14 |
| *LTBP3* | 0.25 | 1.2E-06 |
| *MAP3K7* | 0.16 | 0.0004 |
| *MSTN* | 0.12 | 0.001 |
| *MTMR4* | 0.40 | 7.7E-08 |
| *MYOCD* | 0.19 | 5.7E-07 |
| *PBLD* | 0.05 | 0.009 |
| *PMEPA1* | -0.27 | 0.0009 |
| *PML* | -0.04 | 0.001 |
| *PPM1A* | 0.17 | 0.0009 |
| *RASL11B* | 1.72 | 1.0E-22 |
| *RNF111* | 0.11 | 0.023 |
| *SDCBP* | -0.44 | 9.5E-08 |
| *SIRT1* | 0.24 | 9.2E-05 |
| *SMAD5* | 0.15 | 0.003 |
| *SMAD7* | 0.13 | 0.008 |
| *SMAD9* | 0.22 | 6.5E-07 |
| *SOX11* | -0.31 | 1.72E-07 |
| *TGFB1I1* | -0.22 | 0.00003 |
| *TGFB2* | 0.18 | 0.0138 |
| *TGFBR2* | -0.54 | 6.6E-19 |
| *TGFBR3* | -0.65 | 1.1E-14 |
| *THBS1* | -1.04 | 2.5E-07 |
| *TP53* | -0.13 | 0.0001 |
| *TRIM33* | 0.27 | 1.3E-09 |
| *UBB* | 0.38 | 1.0E-10 |
| *UBC* | 0.16 | 0.002 |
| *USP15* | 0.24 | 0.0002 |
| *USP9X* | 0.11 | 0.006 |
| *VASN* | -0.39 | 1.1E-06 |
| *ZEB1* | 0.48 | 1.1E-13 |
| *ZMIZ1* | 0.21 | 0.0007 |
| *ZMIZ2* | -0.18 | 8.1E-08 |
| *ZNF451* | 0.13 | 0.0001 |
| *ZNF703* | -0.09 | 1.5E-05 |
| *ZYX* | -0.82 | 2.3E-19 |

Benjamini-Hochberg method was used to adjust pvalues for multiple comparisons.(n= 105 HCM patients, n = 39 controls). Genes with log2FC >1 or <-1 are indicated by grey shading.

**SUPPLEMENTARY METHODS**

***Experimental animals:*** All procedures involving the handling of animals were approved by the Animal Care and Use Committee of the Johns Hopkins University and the University of California San Francisco and adhered to the National Institutes of Health Public Health Service guidelines. Transgenic male C57BL/6 mice expressing the R403Q mutation in α-Myosin Heavy Chain (*Myh6 gene*) were kindly provided by Dr. Leinwand. The transgene coding region consists of rat *α*-MyHC cDNA expressing a point mutation (G1445A, resulting in Arg403Gln) and a deletion (of amino acids 468–527) bridged by the addition of nine non-myosin amino acids. The R403Q-αMyHC mouse was bred on a CBA/B16 (F1) cross background^1^. Transgenic male C57Bl/6N mice bearing a c-myc-tagged murine cardiac troponin T (*Tnnt2 gene*) with the R92W mutation were kindly provided by Dr. Tardiff. The R92W-TnT mouse is an F1 cross between FVB/N and C57/Bl6 strains^2,3^. The R403Q-αMyHC and R92W-TnT mice were backcrossed to C57Bl/6 for 5-10 generations before use as breeders^1,2,4^. Male mice were weaned and genotyped at the age of 4 weeks by PCR-amplified tail DNA. DNA was extracted from mouse tail using the Dneasy Blood &Tissue kit (Cat No: 69506, QIAGEN, MD, USA) according to the manufacturer’s instructions. PCR was performed using one *Taq*Hot Start Master Mix (Cat #: M0484S, New England Biolabs, MA, USA), using the following primer sequences:

Housing keeping gene: forward primer (TGAGGTTGTCTTCTGATCTGC), reverse primer (TCCTGGACAAAGTAACCCTTG);

R403Q-MyHC: forward primer (CCAGGTCAACAAGCTGCGG), reverse primer (TGTGGTGTAAATAGCAAAGC);

R92W-TnT: forward primer (ACCTAGAGGGAAAGTGTCTT), reverse primer (TCCTCTTCAGAGATGAGCTTT).

All studies were performed at 24 weeks of age, based on previous studies which revealed the development of a cardiac HCM phenotype at this age^1,2,4^. Euthanasia of the mice was performed using cervical dislocation by trained individuals, prior to harvesting the hearts.

***Echocardiography:*** All images were acquired using a Vevo 3100 imaging ultrasound machine using a MX550D probe (40MHz, VisualSonics, Toronto, Canada) with continuous ECG monitoring. Mice were anesthetized in a plexiglas box using isoflurane 2%, weighed and then placed on a heating pad under a heating lamp to maintain body temperature at 37°C, which was monitored with a rectal thermometer. Isoflurane was titrated to <1% during image acquisition, resulting in heart rates of approximately 500-550 bpm. Cardiac morphology and function were evaluated from conventional M-mode and 2D images using Vevo LAB software. M-mode parasternal long axis images of the left ventricle were acquired at the level of the papillary muscles. At least three consecutive cardiac cycles were analyzed. The investigator performing image analysis was blinded to mutation status of the mice.

***Histology:*** Mice were euthanized using 4% isoflurane. Hearts were extracted, washed in PBS, followed by fixation with 10% formalin for 48 hours and then embedded in paraffin prior to sectioning (5 μm sections). Sirius Red staining was performed to assess collagen content of myocardium. Briefly, 5 μm sections were deparaffinized with xylene, rehydrated using graded concentrations of ethanol (100, 95 and 80%), and counterstained for 25 min with 0.1% Fast Green (Sigma Aldrich), followed by Sirius Red (Sigma Aldrich) for 30 minutes prior to mounting in Permount (Fisher). Images were acquired using a Leica DM5000 B microscope equipped with a Leica DFC7000 T camera (Leica Microsystems GmbH, Wetzlar, Germany). To quantify collagen deposition, 20 fields were randomly selected from the septum, LV apex and lateral wall of each heart (n=2 in each group). The red pixel content of digitized photos was measured relative to total tissue (red and green pixels) using Image J (version 1.52, National Institutes of Health, Bethesda, MD, USA).

***RNA-seq and analysis in 24 week mouse hearts:*** Whole heart total RNA was prepared from three biological replicates for each genotype using miRNeasy (Qiagen). Three biological replicates each of stand-specific, poly-A+ RNA-seq libraries were constructed as described previously^5-8^. Briefly, poly-A+ RNA was purified using Dynabeads Oligo(dT) 25 (Thermo Fisher Scientific), treated with DNase, and partially fragmented at 94 °C. Fragmented RNA was reverse-transcribed to ssDNA, which was then converted to dsDNA in the presence of dUTP. dsDNA was end-repaired, A-tailed, adapter-ligated, and treated with Uracil-DNA Glycosylase treatment. The resulting ssDNA was used as PCR templates to make library dsDNAs. Paired-end 100 nt sequencing (2x 100 bp) was performed using HiSeq2500 platform (Illumina) and analyzed as previously described^7,8^. Approximately 9-15 million paired-end fragment reads were obtained for each library, of which ~97-99% of were mapped to the mm10 mouse genome. MiRNA-seq (small RNA-seq) libraries were prepared, sequenced on HiSeq2500 (Illumina), and analyzed as previously described^5-7,9,10^. Briefly, 18-30 nt long RNAs were size-selected by gel purification and ligated with 3′ adapter and 5′ adapter. The RNAs were reverse-transcribed into ssDNA, which was then used as PCR templates to make library dsDNAs.Approximately 9-29 million reads were obtained for each library, of which ~45-54% were mapped to the mm10 mouse genome, of which ~12-24% were mapped to miRNA hairpins.The SRA accession number for the mRNA-seq and miRNA-seq libraries reported in this manuscript is PRJNA559482**.**

***Human HCM data analysis:*** Gene expression data from publicly available microarray datasets, GSE36961 (mRNA) and GSE36946 (miRNA) analyzed for differential gene expression. The microarray datasets were obtained from ventricular septal tissue of HCM patients undergoing myectomy at the Mayo Clinic (Rochester, MN)^11^, and from age/sex-matched donor hearts (control subjects) from the Sydney Heart Bank^12^. In the control subjects, tissue was obtained either from left ventricular septum or free wall. Demographic information of control subjects and HCM patients, as well as data on HCM patient genotype/phenotype was obtained from the Master’s Thesis by Virginia Hebl^13^.

Briefly, the raw data underwent quality control analysis. The fluorescence signal values were log2 converted and quantile normalized.A microarray batch effect was noted in the data and adjusted for in the subsequent expression analysis. Data from one HCM patient that proved to be an outlier by principle component analysis (PCA), was excluded from further analysis^14^.

For Ingenuity pathway analysis of human samples, mRNA data from 105 HCM patients was compared to 39 controls by ANOVA using the Partek Genomics Suite 7.0 platform. Genes with log2FC greater than 2SD up or down were used in the IPA analysis.A p<0.05 was considered statistically significant.

***Adult mouse myocyte isolation:*** Cardiac myocytes were isolated from 24 week old mouse hearts, using established protocols^5,15,16^. Briefly, mice were heparinized (100 IU heparin) 10 min prior to cervical dislocation. The hearts were rapidly excised, cannulated via the aorta, and perfused in the langendorf mode with a constant perfusion pressure of 80 mm Hg. The hearts were then perfused for 10 min using Ca^2+^-free Tyrode containing (in mM) NaCl (120), KCl (5.4), NaH_2_PO_4_ (1.2), NaHCO_3_ (20), MgCl_2_ (1.6), glucose (1 mg/ml), 2, 3-butanedione monoxime (BDM, 1 mg/ml), taurine (0.628 mg/ml), 0.9 mg/ml collagenase type 2 (Worthington Biochemical Co., 299 U/mg), and gassed with 95% O_2_–5% CO_2_. The heart was cut into small pieces, which were gently agitated, allowing myocytes to be dispersed in the Ca^2+^-free Tyrode containing BSA (5 mg/L) for 10 min. Dispersed myocytes were filtered through a 150 μm mesh and gently centrifuged at 500 rpm for 30 sec. The cells were re-suspended in Ca^2+^-containing gradually increasing Ca^2+^ concentrations (0.125 to 1 mM Ca^2+^), and stored in 1 mM Ca^2+^Tyrode until use.

***Two photon microscopy to assess redox status in isolated mouse cardiac myocytes:*** Experiments were performed at 37°C in a thermostatically controlled flow chamber mounted on the stage of an upright microscope (Nikon E600FN) attached to a multi-photon laser scanning system with excitation at 740 nm^5,15-17^. Cells were suspended in Tyrode solution, pH 7.4, containing (in mM), NaCl (140), KCl (5), MgCl2 (1), HEPES (10), CaCl_2_ (1), and glucose (10). TMRM (tetramethylrhodamine methyl ester, 100 nM, red λ_em_ 605 ± 25 nm) and MCB (monochlorobimane, 50 µM, blue λ_em_ 480 ± 20nm) were loaded for 20 min on the stage of the microscope at 37°C to simultaneously monitor mitochondrial (ΔΨ_m_) and reduced glutathione (GSH) respectively. Autofluorescence of NAD(P)H, namely total fluorescence collected at <490 nm, was monitored separately. The acquired signal was calibrated by the addition of potassium cyanide (KCN, 1mM)for maximum reduction of existing NAD(P)H, followed by addition of trifluoromethoxy carbonylcyanide phenylhydrazone (FCCP, 5 μM) for maximum oxidation of NADPH. All cells were imaged at resting condition (non-beating). Image analysis was performed using Image J software.

***Quantification of mitochondrial DNA copy number:*** Total nucleic acids were extracted from hearts using the Tissue Lyzer disruption system (Qiagen). The homogenate was treated with 1.6 mg/ml RNase A (Qiagen) for 10 minutes at room temperature and then with proteinase K for 20 min at 55 ºC. In order to purify total heart DNA, the digests were mixed with ethanol and then loaded on DNeasy columns, using the manufacturer’s protocol. DNA concentration was quantified using a spectrophotometer (Nanodrop, Thermo Fisher Scientific). Twenty nanograms of DNA were used as template in Taqman-based quantitative real-time PCR. The gene-specific assay for mtDNA was performed using murine cytochrome-c oxidase subunit 1 (COX-I; Cat. # Mm04225243_g1, Life Technologies), a mitochondrial gene, and glyceraldehyde phosphate dehydrogenase (GAPDH, Cat.# Mm99999915_g1), a nuclear gene as described previously^5,18^. The amount of COX-I relative to GAPDH was calculated according to the ΔCt method and normalized (ΔΔCt) to littermate control heart values to obtain a measure of mitochondrial DNA in each samples.

***Mitochondrial Isolation:*** Isolation and handling of mitochondria was performed as previously described^5,17^. Briefly, mice were euthanized by cervical dislocation, hearts were harvested and immersed in ice cold isolation solution (IS, pH 7.4, containing [in mM] Sucrose [75], Mannitol [225], EGTA [1]). Ventricles were homogenized in IS with the addition of 0.1 mg/ml bacterial proteinase (type XXIV, Sigma-Aldrich), followed by 0.2% albumin (fatty acid-free) to block proteinase activity. Homogenate was then centrifuged at 500 g for 10 min to discard unbroken tissue and debris. The supernatant was centrifuged at 10,000 g for 10 min to sediment the mitochondria and then washed twice using IS by centrifuging at 7,700 gfor 5 min. The mitochondrial pellet was re-suspended in Suspension Solution (IS without EGTA) and protein concentration was determined using the bicinchoninic acid method (BCA protein assay kit, Thermo Fisher Scientific).

***Measurement of mitochondrial respiration:***Respiration was evaluated in freshly isolated mitochondria using an automated 96-well extracellular flux analyzer (Seahorse XF96; Seahorse Bioscience, Billerica, MA) and Buffer B (pH 7.2) containing (in mM) KCl (137), KH_2_PO_4_ (2), EGTA (0.5), MgCl_2_ (2.5), HEPES (20) with 0.2% fatty acid-free BSA^5,16^. Mitochondria were assayed in polyethyleneimine-coated XF96 plates. After removing the polyethyleneimine (1:15,000 dilution in buffer B, overnight incubation at 37°C without CO_2_), 10 µg of mitochondrial protein was transferred to each well and centrifuged at 3,000 g for 7 min at 4^o^C, before starting the assay. Mitochondrial respiration from substrates of Complex I was evaluated by robotic injection of 5 mM each of glutamate and malate (GM) to determine State 4 respiration; 1 mM ADP was added to assess State 3 respiration. Succinate (5mM) with Rotenone (1μΜ) followed with/without ADP was used to evaluate Complex II respiration and TMPD (N,N,N′,N′-Tetramethyl-p-phenylenediamine, 0.5 mM) for Complex IV respiration.

***Measurement of mitochondrial membrane potential (ΔΨm), ROS generation and ROS scavenging capacity:***NAD(P)H redox status, mitochondrial swelling and Δψ_m_ were monitored simultaneously using a wavelength-scanning fluorometer (QuantaMaster; Photon Technology International, Inc.) and multidye program^5,17^. Isolated mitochondria were suspended in buffer B (same as above, without BSA). For each assay, 150 µg of mitochondrial protein was suspended in 2 ml of buffer B in a quartz cuvette with a stirring bar at 37 ^o^C. NAD(P)H autofluorescence (*λ*_exc:_340, *λ*_em_:450 nm) and mitochondrial swelling (90° light scattering λ_exc_=520, λ_em_:585 nm) were monitored. NAD(P)H signal was calibrated with the addition of KCN (2.5 mM) for maximal reduction and 2,4-dinitrophenol (DNP, 20 μΜ) for minimal reduction. The % of NAD(P)H reduced was then estimated during State 4 and State 3. Δψm was recorded using tetramethylrhodamine methyl ester (TMRM; 100 nM) by applying the ratiometric method of Scaduto and Grotyohann^19^ which uses *λ*_exc_: 546 nm and 573 nm, and *λ*_em_: 590 nm.

Amplex Red (AR) from Life Technologies was used to measure H_2_O_2_(ROS) production by isolated mitochondria^5,17^. Mitochondrial suspensions (150 ug) with added 10 µM AR and Horseradish peroxidase (1 U/ml, HRP, Sigma) were loaded in 2 ml Buffer B, in a quartz cuvette with a stirring bar at 37 ^o^C. Resorufin, the fluorescent product obtained by the 1:1 stoichiometric oxidative reaction of horseradish conjugated-AR with H_2_O_2_ was monitored at *λ*_exc_=530 nm and *λ*_em_= 590 nm. Glutamate/malate (5 mM) was used to measure ROS generation via Forward Electron Transport (NADH dependent respiration, through Complex I) and succinate (5 mM) + rotenone (1 μM, Complex I inhibitor) was used to assess Complex I Reverse Electron Transport. Glutamate/malate (5/5 mM) or succinate (5 mM) were used to measure ROS generation during state 4 respiration; ADP (1 mM) was added to measure ROS generation during state 3 respiration. At the end of the experiment, calibration of the AR signal was achieved with 100 picomoles H_2_O_2_. Quantification of the H_2_O_2_ produced was based on estimation of the slope of the AR signal. [H_2_O_2_] (during state 4 or state 3 respiration)= slope of AR (during state 4 or 3) *100(pmoles)/calibration slope/mitochondrial protein used x 60seconds/duration of measurement (state 4 or 3), as previously described^17^. In order to evaluate ROS scavenging capacity by the glutathione and thioredoxin systems in isolated mitochondria^5,16^, Auranofin (50 nM, Sigma) and dinitrochlorobenzene (DNCB, 10 µM, Sigma) were used to inhibit selectively the thioredoxin and glutathione systems respectively in the inter-membrane space and mitochondrial matrix, while H_2_O_2_ emission was recorded. Initially both inhibitors were used in order to achieve maximum ROS emission, with no scavenging. Then, selective blockade with each inhibitor permitted estimation of the extent of scavenging by each system.

***Measurement of mitochondrial calcium handling:*** Mitochondria (~600μg) were suspended in a buffer (pH 7.2) containing (in mM) KCl (137), KH_2_PO_4_ (2), EGTA (0.02) and HEPES^5,20^. Extra-mitochondrial and intra-mitochondrial [Ca^++^] were measured simultaneously using Calcium green-5N (0.1 μM, hexopotassium salt, cell impermeant, Life Technologies; *λ*_exc_:505, *λ*_em_:535nm) and Fura-FF(*λ*_exc_:340 and 380nm, *λ*_em_:510nm), respectively. In order to quantify mitochondrial matrix [Ca^++^]_free_, isolated mitochondria were loaded with Fura-FF (20 μM incubation for 30 min at room temperature followed by washing 2-3 times with SS as described above). The Fura-FF signal was calibrated by treating mitochondria with the Ca^++^ionophore 4-bromo-A23187 (2 μM), oligomycin (5 μg/ml) and FCCP (5 μM) to allow equilibration between intra- and extra-mitochondrial Ca^++^. The calibration curve was established according to the equation: [Ca^2+^]=K_d_’β (R- R_min_)/( R_max_ -R) where R is the ratio of *λ*_em_:510nm intensities for *λ*_exc_: 340 and 380 nm. K_d_’ is the apparent Ca-fura-FF dissociation constant, and β is the fluorescence intensity ratio for Ca^++^-free and Ca^++^-saturated fura-FF excited at 380 nm. R_max_ and R_min_ are R values for Ca^++^-saturated and Ca^++^-free Fura-FF. The experiment was performed in energized mitochondria (at State 4, with GM) at room temperature. Repeated additions of [Ca^+2^] (5 μM) were performed at 1 min intervals. Total [Ca^+2^] until PTP opened and matrix [Ca^++^]_free_ were calculated using the online version of WEBMAXC (http://maxchelator.stanford.edu/webmaxc/webmaxcE.htm). Mitochondrial permeability transition pore opening was characterized by abrupt collapse of Δψ_m_, swelling and changes in recorded [Ca^++^], namely, a decrease in the Fura-FF signal and increase in the Ca Green signal.

**References**

1 Vikstrom, K. L., Factor, S. M. & Leinwand, L. A. Mice expressing mutant myosin heavy chains are a model for familial hypertrophic cardiomyopathy. *Molecular medicine (Cambridge, Mass.)* **2**, 556-567 (1996).

2 Ertz-Berger, B. R. *et al.* Changes in the chemical and dynamic properties of cardiac troponin T cause discrete cardiomyopathies in transgenic mice. *Proceedings of the National Academy of Sciences of the United States of America* **102**, 18219-18224, doi:10.1073/pnas.0509181102 (2005).

3 Tardiff, J. C. *et al.* Cardiac troponin T mutations result in allele-specific phenotypes in a mouse model for hypertrophic cardiomyopathy. *The Journal of clinical investigation* **104**, 469-481, doi:10.1172/jci6067 (1999).

4 Geisterfer-Lowrance, A. A. *et al.* A mouse model of familial hypertrophic cardiomyopathy. *Science (New York, N.Y.)* **272**, 731-734 (1996).

5 Vakrou, S. *et al.* Allele-specific differences in transcriptome, miRNome, and mitochondrial function in two hypertrophic cardiomyopathy mouse models. *JCI Insight* **3**, doi:10.1172/jci.insight.94493 (2018).

6 Liao, S. E., Ai, Y. & Fukunaga, R. An RNA-binding protein Blanks plays important roles in defining small RNA and mRNA profiles in Drosophila testes. *Heliyon* **4**, e00706, doi:10.1016/j.heliyon.2018.e00706 (2018).

7 Zhu, L., Kandasamy, S. K., Liao, S. E. & Fukunaga, R. LOTUS domain protein MARF1 binds CCR4-NOT deadenylase complex to post-transcriptionally regulate gene expression in oocytes. *Nature communications* **9**, 4031, doi:10.1038/s41467-018-06404-w (2018).

8 Liao, S. E., Kandasamy, S. K., Zhu, L. & Fukunaga, R. DEAD-box RNA helicase Belle posttranscriptionally promotes gene expression in an ATPase activity-dependent manner. *RNA (New York, N.Y.)* **25**, 825-839, doi:10.1261/rna.070268.118 (2019).

9 Kandasamy, S. K. & Fukunaga, R. Phosphate-binding pocket in Dicer-2 PAZ domain for high-fidelity siRNA production. *Proceedings of the National Academy of Sciences of the United States of America* **113**, 14031-14036, doi:10.1073/pnas.1612393113 (2016).

10 Kandasamy, S. K., Zhu, L. & Fukunaga, R. The C-terminal dsRNA-binding domain of Drosophila Dicer-2 is crucial for efficient and high-fidelity production of siRNA and loading of siRNA to Argonaute2. *RNA (New York, N.Y.)* **23**, 1139-1153, doi:10.1261/rna.059915.116 (2017).

11 Bos, J. M. *et al.* Marked Up-Regulation of ACE2 in Hearts of Patients With Obstructive Hypertrophic Cardiomyopathy: Implications for SARS-CoV-2-Mediated COVID-19. *Mayo Clin Proc* **95**, 1354-1368, doi:10.1016/j.mayocp.2020.04.028 (2020).

12 Dos Remedios, C. G. *et al.* The Sydney Heart Bank: improving translational research while eliminating or reducing the use of animal models of human heart disease. *Biophysical reviews* **9**, 431-441, doi:10.1007/s12551-017-0305-3 (2017).

13 Hebl, V. *The messenger RNA and microRNA transcriptomes of hypertrophic cardiomyopathy*, (2012).

14 Liu, Y. *et al.* Differences in microRNA-29 and Pro-fibrotic Gene Expression in Mouse and Human Hypertrophic Cardiomyopathy. *Frontiers in cardiovascular medicine* **6**, 170, doi:10.3389/fcvm.2019.00170 (2019).

15 Tocchetti, C. G. *et al.* Nitroxyl improves cellular heart function by directly enhancing cardiac sarcoplasmic reticulum Ca2+ cycling. *Circulation research* **100**, 96-104, doi:10.1161/01.RES.0000253904.53601.c9 (2007).

16 Tocchetti, C. G. *et al.* GSH or palmitate preserves mitochondrial energetic/redox balance, preventing mechanical dysfunction in metabolically challenged myocytes/hearts from type 2 diabetic mice. *Diabetes* **61**, 3094-3105, doi:10.2337/db12-0072 (2012).

17 Aon, M. A., Cortassa, S. & O'Rourke, B. Redox-optimized ROS balance: a unifying hypothesis. *Biochimica et biophysica acta* **1797**, 865-877, doi:10.1016/j.bbabio.2010.02.016 (2010).

18 Papanicolaou, K. N. *et al.* Mitofusins 1 and 2 are essential for postnatal metabolic remodeling in heart. *Circulation research* **111**, 1012-1026, doi:10.1161/circresaha.112.274142 (2012).

19 Scaduto, R. C., Jr. & Grotyohann, L. W. Measurement of mitochondrial membrane potential using fluorescent rhodamine derivatives. *Biophysical journal* **76**, 469-477, doi:10.1016/s0006-3495(99)77214-0 (1999).

20 Wei, A. C., Liu, T., Cortassa, S., Winslow, R. L. & O'Rourke, B. Mitochondrial Ca2+ influx and efflux rates in guinea pig cardiac mitochondria: low and high affinity effects of cyclosporine A. *Biochimica et biophysica acta* **1813**, 1373-1381, doi:10.1016/j.bbamcr.2011.02.012 (2011).
